# Neurotoxic effects in zebrafish embryos by valproic acid and nine of its analogues: the fish-mouse connection?

**DOI:** 10.1007/s00204-020-02928-7

**Published:** 2020-10-27

**Authors:** Katharina Brotzmann, André Wolterbeek, Dinant Kroese, Thomas Braunbeck

**Affiliations:** 1 Aquatic Ecology and Toxicology Group, Centre for Organismal Studies, University of Heidelberg, Im Neuenheimer Feld 504, 69120 Heidelberg, Germany; 2 TNO Healthy Living Unit, Department of Risk Analysis for Products in Development, The Netherlands Organization for Applied Scientific Research, Princetonlaan 6, 3584 CB Utrecht, The Netherlands

**Keywords:** Zebrafish, Embryo, Neural tube defects, Neurotoxicity, Valproic acid, Analogues, Correlation fish/mouse

## Abstract

Since teratogenicity testing in mammals is a particular challenge from an animal welfare perspective, there is a great need for the development of alternative test systems. In this context, the zebrafish (*Danio rerio*) embryo has received increasing attention as a *non-protected* embryonic vertebrate in vivo model. The predictive power of zebrafish embryos for general vertebrate teratogenicity strongly depends on the correlation between fish and mammals with respect to both overall general toxicity and more specific endpoints indicative of certain modes-of-action. The present study was designed to analyze the correlation between (1) effects of valproic acid and nine of its analogues in zebrafish embryos and (2) their known neurodevelopmental effects in mice. To this end, zebrafish embryos exposed for 120 h in an extended version of the acute fish embryo toxicity test (FET; OECD TG 236) were analyzed with respect to an extended list of sublethal endpoints. Particular care was given to endpoints putatively related to neurodevelopmental toxicity, namely jitter/tremor, deformation of sensory organs (eyes) and craniofacial deformation, which might correlate to neural tube defects caused by valproic acid in mammals. A standard evaluation of lethal (LC according to OECD TG 236) and sublethal toxicity (EC) merely indicated that four out of ten compounds tested in zebrafish correlate with positive results in mouse in vivo studies. A detailed assessment of more specific effects, however, namely, jitter/tremor, small eyes and craniofacial deformation, resulted in a correspondence of 75% with in vivo mouse data. A refinement of endpoint analysis from an integration of all observations into one LC_x_ or EC_x_ data (as foreseen by current ecotoxicology-driven OECD guidelines) to a differential evaluation of endpoints specific of selected modes-of-action thus increases significantly the predictive power of the zebrafish embryo model for mammalian teratogenicity. However, for some of the endpoints observed, e.g., scoliosis, lordosis, pectoral fin deformation and lack of movement, further experiments are required for the identification of underlying modes-of-action and an unambiguous interpretation of their predictive power for mammalian toxicity.

**Electronic supplementary material:**

The online version of this article (10.1007/s00204-020-02928-7) contains supplementary material, which is available to authorized users.

## Introduction

Given an ever-growing number and amount of industrial chemicals, pesticides, biocides, drugs and cosmetics, a comprehensive and highly integrated regulatory system for hazard and risk assessment of chemical substances has been installed worldwide to protect the health of humans and the environment (OECD 2014; Scholz et al. 2013). This has led to a considerable increase in the numbers of animals used for toxicity testing, and in 2017 alone, more than 2,180,000 animals (172,000 fish) were used for regulatory toxicological and other safety assessments in the EU (European-Commission 2020), which does not even include tests conducted outside of Europe for purposes of meeting European chemical legislation. These numbers account for 14% of all animals used for research and testing in the EU in 2017. This has given rise to significant ethical concern not only in the scientific community, but in the entire society, and—at least in the EU—modern legislations for chemical control such as REACH (Registration, Evaluation, Authorization and Restriction of Chemicals) (EU 2006) in conjunction with the current EU Animal Welfare Regulation (EU 2010) provide a clear mandate to develop and implement alternative methods to account for the 3Rs principle by Russell and Burch (1959). Since especially the assessment of reproductive, developmental and ecotoxicity testing is still associated with moderate (if not severe) suffering of the experimental animals (European Commission, 2020), there is an urgent need for the development of alternative methods in toxicity and ecotoxicity testing.

Within the EU-funded consortium EU-ToxRisk, a large integrated European in vitro “flagship” toxicology project exploring new alternative-to-animal approaches to chemical safety evaluation (Daneshian et al. 2016; Escher et al. 2019; Leist et al. 2017), one project addresses alternatives to mammalian teratogenicity testing, which, especially from an animal welfare point of view, represents a major challenge. One alternative approach to mammalian testing is based on the use of early developmental stages of lower vertebrates (fish), which are not regarded protected according to current EU animal welfare legislation (EU 2010; Strähle et al. 2012). Over the last two decades, the zebrafish (*Danio rerio*) embryo has developed into one of the most promising models not only in ecotoxicity testing, but also in mammalian toxicology (Ali et al. 2011b; Bambino and Chu 2017; Brannen et al. 2010; Braunbeck 2009; de Esch et al. 2012; Driessen et al. 2013; Fernandes et al. 2017; Guo et al. 2015; Kari et al. 2007; Nishimura et al. 2015; Scholz 2013; Sipes et al. 2011; Sukardi et al. 2011; Ton et al. 2006; Weigt et al. 2011). As a small cyprinid, zebrafish is not only inexpensive, easy to maintain and to breed in large numbers, but also provides fully transparent embryos, which allow continuous access to developmental disorders in a non-protected model system outside the (mammalian) mother (Braunbeck et al. 2015).

Most importantly, in screening tests, the zebrafish data also showed concordance of at least 80% to mammalian developmental toxicity (Bachmann 2002; Brannen et al. 2010; Nagel 2002) or rodent models and even humans (MacRae and Peterson 2015). Approximately 84% of genes known to be associated with human diseases and a large number of drug metabolism pathways are shared by human and zebrafish or have a zebrafish counterpart (Howe et al. 2013; MacRae and Peterson 2015; Uechi and Kenmochi 2019), and about 70% of human genes have at least one obvious zebrafish orthologue (Howe et al. 2013). Furthermore, the types of effects observed in zebrafish could frequently be correlated with those found in mammals, which indicates the utility and efficiency of the zebrafish embryo model for the detection of at least strong mammalian toxicants (Ball et al. 2014; Brannen et al. 2010; Iida et al. 2019; Kim et al. 2011).

In the present experiment, zebrafish embryos were screened for the teratogenic potential of valproic acid (VPA), an antiepileptic drug, and several chemically related substances (analogues), which were suspected to produce hazardous effects in humans similar to those by VPA (Herrmann 1993). In fact, previous comparisons of effects by anticonvulsants in zebrafish with mammalian in vivo data and human clinical data showed a promising correlation of up to 88% (Berghmans et al. 2007; Lee et al. 2013; Nagel 2002). However, only Nau and Löscher (1986) attempted to discriminate between VPA and several of its analogues by testing them also in mice. The same approach was taken in zebrafish embryos by Herrmann (1993), who, however, restricted himself to an assessment of summary toxicity of the antiepileptics, but ignored any more specific effects such as neural tube defects, which are among the most common and most severe disorders of fetuses and neonates in mammalian species after exposure to VPA (Duru and Ceylan 2019; Hill et al. 2010; Nau 1986; Nau et al. 1991).

For this reason, the focus of the present study was laid on a comparison of indicators of general toxicity by VPA and nine of its analogues (Table 1) in zebrafish embryos with an analysis of more specific endpoints that were selected for their potential correspondence with changes associated with symptoms of neural tube defects in mammals. For the selection of specific endpoints, fundamental differences in neurulation between zebrafish and mammals had to be considered: The development of the neural tube can be categorized into primary and secondary neurulation. During *primary neurulation*, the cells surrounding the neural plate direct the neural plate cells to proliferate, invaginate, and pinch off from the surface to form a hollow tube (Gilbert 2000; Yuskaitis and Pomeroy 2017). In contrast, in *secondary neurulation*, a solid cord of cells along the dorsal axis of the embryo sinks down and subsequently cavitates to form a hollow tube (Gilbert 2000; Yuskaitis and Pomeroy 2017). Since secondary neurulation is based on the migration of individual cells (Ahsan et al. 2019; Araya et al. 2016; Hiscock et al. 2018) and not on folding of complete epithelia, such processes cannot be visualized by simple morphological techniques, i.e., without specific tagging of individual cells.

**Table 1:** Chemical identity and test concentrations of valproic acid and its analogues tested in the Fish Embryo Acute Toxicity test with zebrafish (*Danio rerio*)

Compound	Chemical structure	CAS	Mol. weight (g/mol)	*K*_OW_	Nominal concentration	
					µM	mg/L
2,2-Dimethylvaleric acid		1185-39-3	130.19	2.43	267 321 385 462 555 666 800	35 42 50 60 72 87 104
2-Ethylbutyric acid		88-09-5	116.16	1.68	100 200 400 800 1000	12 23 46 93 116
2-Ethylhexanoic acid		149-57-5	144.21	2.64	6.25 12.5 25 50 100 200 400	1 2 4 7 14 29 58
Hexanoic acid		142-62-1	116.16	1.92	512 563 619 681 750 900	60 65 71 79 87 105
2-Methylhexanoic acid		4536-23-6	130.19	2.47	125 250 500 1000	16 33 65 130
2-Methylpentanoic acid		97-61-0	116.16	1.80	177.7 266.6 400 600 900 1350	21 31 46 70 105 157
2-*n*-Propylheptanoic acid		31080-39-4	172.27	3.20	12.5 25 50 100	2 4 9 17
4-Pentenoic acid		591800	100.12	1.42	414 538 700 910 1183 1538 2000	41 54 70 91 118 154 200
4-*ene* Valproic acid		1,575,720	142.20	2.82	79.01 118.5 177.7 266.6 400 600 800	11 17 25 38 57 85 114
Valproic acid (VPA)		99,661	144.21	2.75	6.25 12.5 25 50 100 200 400 800	1 2 4 7 14 29 58 115

In humans, both neurulation processes play a role. More specifically, the process of neurulation can be subdivided into two distinct phases: (a) primary neurulation during weeks 3 and 4 of gestation leading to the development of the brain and spinal cord, and (b) secondary neurulation occurring in weeks 5 and 6, with formation of the lower sacral and coccygeal cord (Gilbert 2000; Greene and Copp 2014; Mitchell et al. 2004; Yuskaitis and Pomeroy 2017). In humans, secondary neurulation only starts when primary neurulation has been completed and the posterior neuropore has been closed, with the latter also being the prime region of interest for spinal cord malformations such as *spina bifida* (Copp et al. 2015; Northrup and Volcik 2000; Yuskaitis and Pomeroy 2017). In fish, neurulation is exclusively secondary (Gilbert 2000; Yuskaitis and Pomeroy 2017), which is also of ecological relevance, since an open neural tube would be absolutely lethal in the non-sterile environment of aquatic ecosystems. Given these fundamental differences in neurulation processes between mammals and fish, specific effects putatively related or surrogate to neural tube defects were selected as endpoints, namely jitter/tremor as behavioral changes resulting from neurotoxicity, as well as deformation of the eye as a major sensory organ and craniofacial deformation as an indicator of changes potentially associated with malformation of the brain.

An in-depth literature search revealed that in vivo developmental data for VPA and its structural analogues are available for various mammalian models, whereas for zebrafish only VPA data could be localized. To fill this gap with experimental data, extended fish embryo acute toxicity tests (FETs) based on OECD TG 236 (OECD 2013) were conducted and analyzed following three different approaches: (1) In accordance with OECD TG 236, a standard analysis of toxicity was carried out using the 4 core endpoints listed in the guideline (coagulation, lack of somite formation, lack of heartbeat, lack of tail detachment) to form one summarizing toxicity value of 50% lethality (LC_50_). (2) In a second approach, the 4 core endpoints of acute lethality were complemented by any other observation indicative of both lethal and sublethal changes (Table 2) to calculate no (NOECs) and lowest observed effect concentrations (LOECs) as well as 10% levels for lethality (LC_10_) and any observed effect (EC_10_). (3) In a third approach, each observation for VPA and its nine analogues was analyzed separately with respect to NOEC, LOEC, EC_10_ and EC_50_ data as well concentration-effect relationships. The latter multi-endpoint approach was expected to result in higher sensitivity and better predictivity of the zebrafish embryo toxicity test for developmental (neuro)toxicity screening (Beker van Woudenberg et al. 2014). By selection of such analyses of increasing specificity, the study was designed to answer the following questions: (1) Which of the selected VPA analogues would also be teratogenic in the zebrafish embryo? Would the zebrafish embryo be able to predict in vivo-negative or in vivo-positive results in mice? (2) Would an isolated analysis of specific endpoints improve the predicative power of testing in zebrafish embryos relative to a simple evaluation based on a summary combination of all effects?

**Table 2:** List of endpoints recorded in the 120 h Fish Embryo Acute Toxicity (FET) tests with zebrafish (*Danio rerio*) embryos

Core endpoints of acute lethality (OECD TG 236; OECD 2013)	Sublethal endpoints (“any other observation”)
Coagulation Lack of somite formation Lack of tail detachment Lack of heartbeat	**Jitter/tremor** **Eye deformation (small eyes)** **Craniofacial deformation** Developmental retardation Spontaneous movement Lack of pigmentation Reduced yolk resorption Reduced heartbeat Blood congestion Formation of edemata (pericardium, yolk) Scoliosis/lordosis Lack of hatch

The lethal endpoints are defined by OECD TG 236 (OECD 2013). The sublethal endpoints specifically analyzed for their relationship to neurotoxicity are given in bold letters

## Materials and methods

### Chemicals

With the exception of 4-*ene* valproic acid (Santa Cruz Biotechnologies, Dallas, Texas, USA), all test chemicals (Table 1) were purchased at the highest purity available (> 98%) from Sigma-Aldrich (Deisenhofen, Germany). The same holds true for all other substances, unless stated otherwise. Test solutions were freshly prepared in dilution water according to annex 2 of OECD TG 203 (OECD 1992) prior to each experiment, and the pH of the dilution water was adjusted using hydrogen chloride and sodium hydroxide before the addition of the test substances. Addition of the test compounds usually resulted in a decline of pH. However, since OECD TG 236 allows for a pH range between 6.5 and 8.5, no further correction of pH was made for the purpose of the present manuscript.

The final concentration ranges of the test compounds are listed in Table 1. Technically, only 2-*n*-propylheptanoic acid required the use of dimethylsulfoxide (DMSO; Grüssing, Filsum, Germany) as a solvent; however, for reasons of comparability, all test compounds were dissolved in 100% DMSO and then diluted with dilution water to a final concentration of 0.1% DMSO. Test solutions were replaced after 24, 48, 72, 96 h of exposure. For validation of the real test concentrations, media samples were analyzed by liquid chromatography and mass spectrometry. Since analyses confirmed most real media concentrations within a range of 30%, test concentrations are reported as nominal concentrations.

### Fish maintenance

Adult zebrafish (*Danio rerio*) of the wild-type strain ‘Westaquarium’ were obtained from in-house breeding facilities of the Aquatic Ecology and Toxicology Group at the Center for Organismal Studies (University of Heidelberg; licensed under no. 35-9185.64/BH). Fish maintenance as well as breeding and spawning conditions were described in detail by Lammer et al. (2009). In brief, a breeding stock of zebrafish aged between 6 and 24 months was used for egg production. Spawners were free from externally visible diseases and had not been treated with any pharmaceutical (acute or prophylactic) for 6 months before spawning. Females and males were kept together in glass aquaria providing sufficient space for swimming (i.e., ≥ 1 L per fish). Standardized dilution water as specified in ISO 7346–1 and 7346–2 (ISO 1996; 294.0 mg/L CaCl_2_ × 2 H_2_O; 123.3 mg/L MgSO_4_ּ × 7 H_2_O; 63.0 mg/L NaHCO_3_; 5.5 mg/L KCl) or suitable drinking water with ≥ 60% oxygen saturation was used for keeping and breeding. Temperature was maintained at 26 ± 1 °C, and fish were kept under a constant artificial dark/light cycle of 10/14 h. Constant filtering or permanent flow-through conditions guarantee that ammonia, nitrite, and nitrate were kept below detection limits (0–5, 0.025–1 and 0–140 mg/L, respectively). Fish were fed a commercially available artificial diet (TetraMin™ flakes; Tetra, Melle, Germany) twice daily, occasionally supplemented with *Artemia* (Sanders Premium Great Salt Lake; Ogden, Utah, USA) nauplii or *Paramecium* protozoans of appropriate size obtained from an uncontaminated source. Overfeeding was strictly avoided to ensure optimal water quality; remaining food and feces were removed daily.

### Exposure of zebrafish embryos

For the Fish Embryo Acute Toxicity (FET) test, egg production was performed according to OECD TG 236 (OECD 2013) with the exception that the duration of the experiments was extended to 120 h, which, however, is still within the developmental phase defined as non-protected (EU 2010) according to Strähle et al. (2012). In brief, freshly fertilized eggs (< 1 h post-fertilization (hpf)) were seeded into 25 ml crystallizing dishes filled with the respective test solution. After fertilization success had been controlled, eggs were individually transferred into 24-well plates (TPP, Trasadingen, Switzerland) with 1 ml of test solution per embryo. All test vessels had been pre-incubated with the test solutions for at least 24 h. Subsequently, well plates were sealed with self-adhesive foil (SealPlate®, Dunn, Asbach, Germany) to prevent evaporation and cross-contamination and placed in a HettCube 600R incubator (Hettich, Tuttlingen, Germany) at 26 ± 1 °C under a 10/14 h dark/light regime. The test medium was renewed each day (semi-static exposure), and lethal and sublethal effects in the embryos were documented at 24, 48, 72, 96 and 120 hpf according to OECD TG 236 (OECD 2013) and Nagel (2002), respectively. FETs with a minimum mortality rate of 30% in the positive control (4 mg/L 3,4-dichloroaniline) and a maximum effect rate of 10% in the negative control (dilution water) at 120 hpf were classified as valid. All tests were run in triplicates.

### Data analysis and scoring of morphological effects

For documentation of morphological alterations, images were recorded on an Olympus CKX41 inverted microscope (Olympus, Hamburg, Germany) and captured using the Olympus C5040 AUD camera (Olympus, Hamburg, Germany).

Lethal (LC) and effect (EC) concentrations were calculated with ToxRat® (vers. 2.10.03; ToxRat Solutions, Alsdorf, Germany), with both lethal and sublethal effects included for the calculation of EC values.

Design of graphs and statistical analyses were performed using SigmaPlot® 13.0 (Jandel-Systat, Erkrath, Germany). Data analysis was accomplished following two separate strategies:

1. In the standard FET approach, all observations made for a certain time-point and a given exposure concentration were taken together as a cumulative data point. In case of the four lethal core endpoints of OECD TG 236 (coagulation, lack of somite formation, lack of tail detachment, lack of heartbeat (blood circulation; Table 2), the cumulative effect was termed “lethal concentration” (LC). In case of all observations, i.e. the lethal four core endpoints plus any other (sublethal) observation, the cumulative effect was termed “effect concertation” (EC).
  Out of the sublethal endpoints, three parameters were specifically analyzed due to their potential relationship to neurotoxicity: jitter/tremor (indicative of modified nerve transmission), eye deformation (small eyes as indicator of malformation of sensory organs) and craniofacial deformation (as a morphological orthologue to neural tube defects in mice (https://aopwiki.org/aops/275) based on inhibition of histone deacetylase (HDAC) (https://aopwiki.org/aops/274) (Gurvich et al. 2005; Kong et al. 2014; Massa et al. 2005; McGee-Lawrence and Westendorf 2011; Menegola et al. 2005; Murko et al. 2013; Pillai et al. 2004; Rao and LaBonne 2018)).
2. In a more detailed approach, using the same software, separate graphs were generated for all effects observed, namely pericardial edemata, yolk edemata, yolk discoloration, yolk sac absorption reduced, yolk sac deformation, reduced heartbeat, lack of heartbeat, blood congestion, scoliosis/lordosis, chorda deformation, loss of somite differentiation, craniofacial deformation, small eyes, eye under-pigmentation, head deformation, reduced otic vesicles, lack of otoliths, enlarged otic vesicles, brain discoloration, body under-pigmentation, jitter/tremor, pectoral fins deformed, lack of movement, lying in a lateral position, lack of hatch. For this purpose, the numbers (x out of 20–40) of embryos exhibiting specific effects were scored, and the resulting concentration-effect graphs were used to compute EC_10_ values (defined as the concentration, where a 10% increase of incidence of a monitored effect over controls could be recorded). EC_10_ values for specific endpoints of all compounds were then used for ranking the compounds with respect to their potency to induce the specific effect in question. As for the standard approach, out of all lethal and sublethal effects, three effects were selected for comparison with known in vivo potencies of mice expressing exencephaly: jitter/tremor, small eyes and craniofacial deformation.

## Results

### General toxicity in the standard fish embryo test

**Fig. 1:**
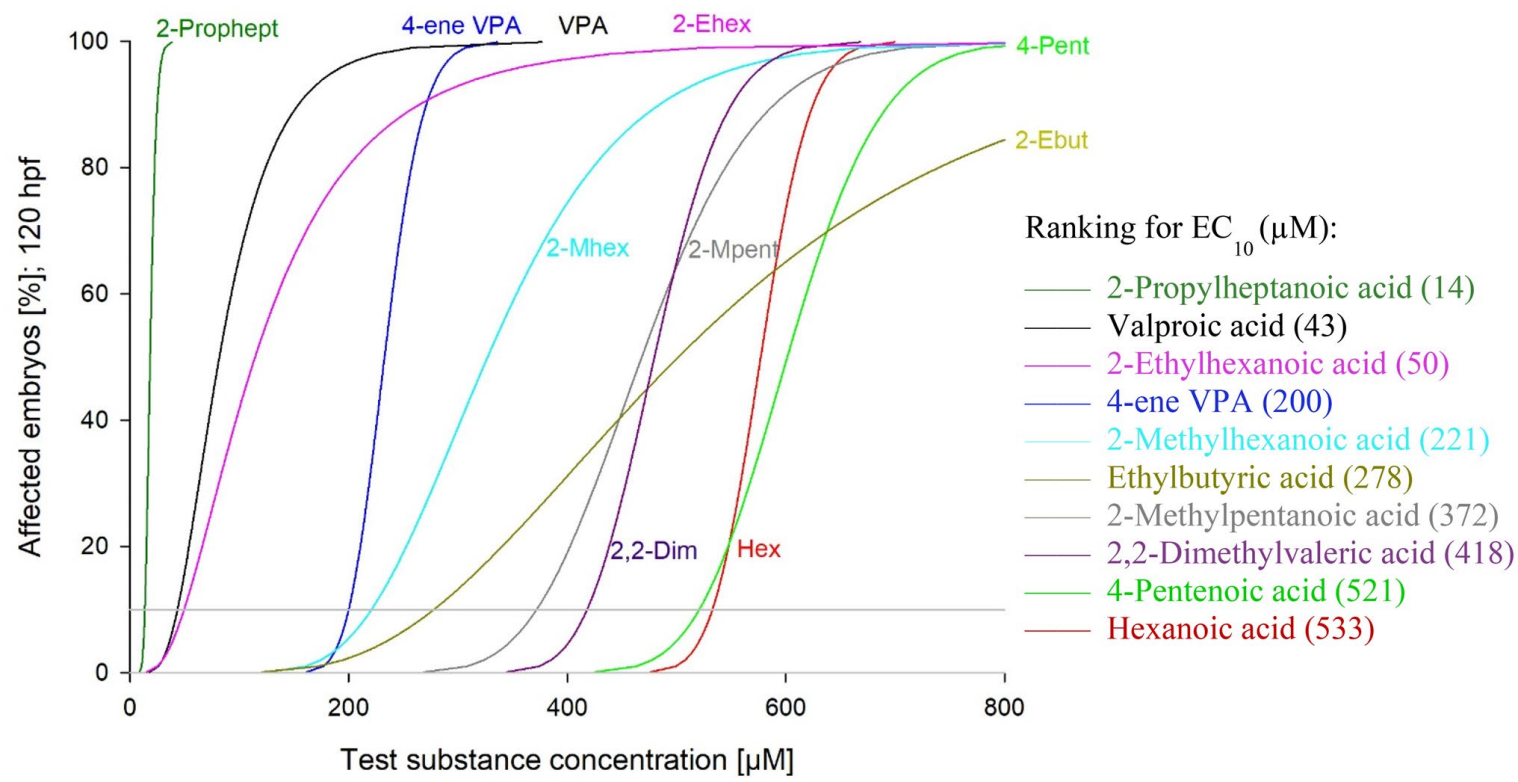
Cumulated portion (% of individuals) of zebrafish (*Danio rerio*) embryos (*n* = 20–40) showing any lethal or sublethal effect after 120 h exposure to valproic acid and its nine analogues. The sequence of the chemicals represents the ranking in overall toxicity. Data in brackets: effective concentrations (µM), where 10% of the embryos show an effect (EC_10_)

In the standard FET, results are given as one single value such as an LC_50_ or an EC_50_ for acutely lethal or sublethal effects, respectively—as holds true for any conventional short-term test in ecotoxicology. Especially, when it comes to more specific modes of action, however, EC_10_ values have received increasing interest in an attempt to exclude interference with systemic toxicity. An alignment of the ten test substances according to their EC_10_ values (Fig. 1) revealed 2-*n*-propylheptanoic acid, valproic acid, 2-ethylhexanoic acid and 4-*ene* valproic acid as the most toxic compounds for fish embryos (Fig. 1, Table 3):

**Table 3:** Cumulative lethal (LC) and sublethal effect concentrations (EC) as well as NOECs and LOECs derived from all lethal and sublethal endpoints in zebrafish (*Danio rerio*) embryos after 120 h of exposure to valproic acid and its nine analogues

Substance	NOEC (µM)	LOEC (µM)	Sublethal effects (µM)		Lethal effects (µM)	
			EC_10_	EC_50_	LC_10_	LC_50_
2-*n*-Propylheptanoic acid	12	13	14	18	52	67
Valproic acid	13	25	43	82	231	435
2-Ethylhexanoic acid	25	50	50	114	168	265
4-*ene* Valproic acid	80	119	200	233	350	485
2-Methylhexanoic acid	125	< 221	221	327	578	646
2-Ethylbutyric acid	100	200	278	502	527	721
2-Methylpentanoic acid	250	267	372	468	450	584
2,2-Dimethylvaleric acid	313	400	418	480	551	593
4-Pentenoic acid	512	520	521	601	634	1034
Hexanoic acid	414	500	533	577	642	665

Data are listed according to scoring for decreasing toxicity

*NOEC* no observed effect concentration, *LOEC* lowest observed effect concentration

2-*n*-propylheptanoic acid > valproic acid > 2-ethylhexanoic acid > 4-*ene* valproic acid > 2-methylhexanoic acid > 2-ethylbutyric acid > 2-methylpentanoic acid > 2,2-dimethylvaleric acid > 4-pentenoic acid > hexanoic acid.

Most interestingly, all of these four substances that showed the highest toxicity in zebrafish embryos also induced exencephaly in mice (Nau and Löscher 1986; Nau and Zierer 1982; Padmanabhan and Ahmed 1996; Paulson et al. 1985; Sonoda et al. 1990). In addition, 4-pentenoic acid as one of the least toxic compounds in the FET had also proved in vivo-negative in mice.

### Toxicity scoring for specific (sublethal) endpoints in the FET

**Fig. 2:**
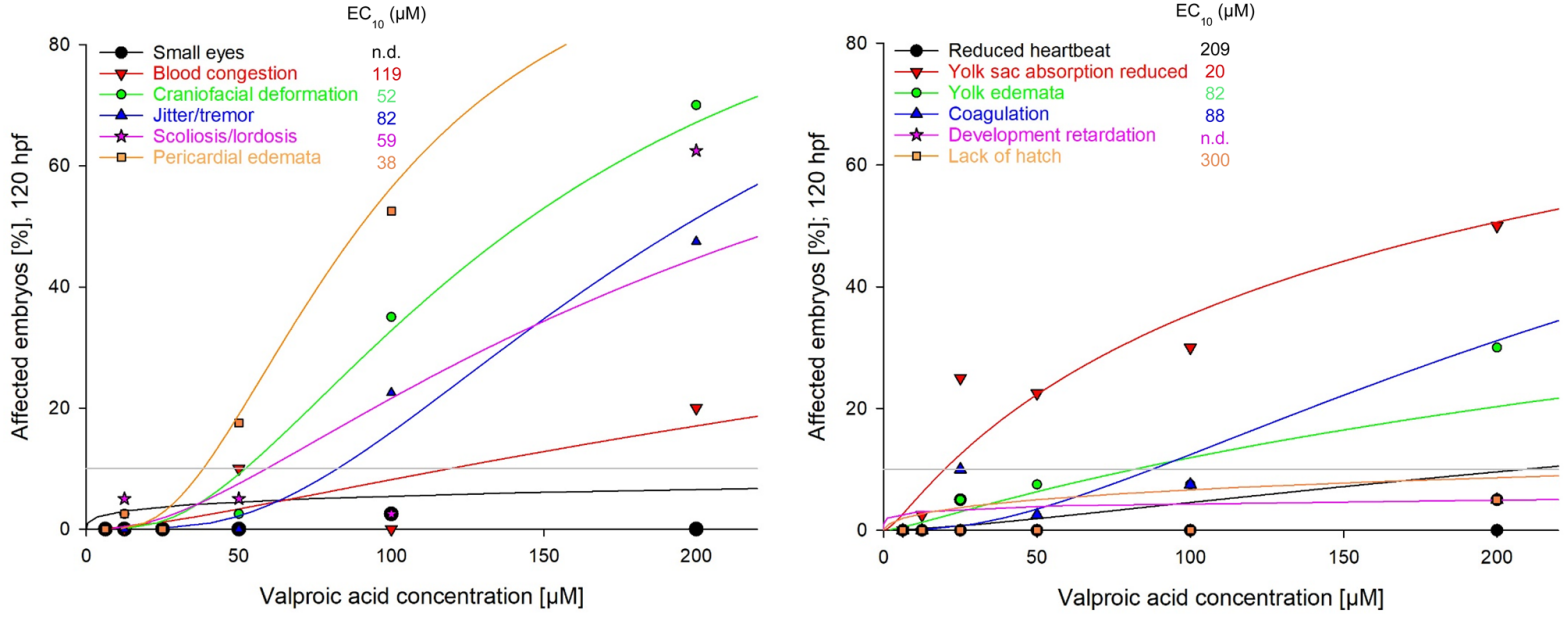
Concentration–response curves and EC_10_ values for specific sublethal endpoints recorded after 120 h exposure of zebrafish (*Danio rerio*) embryos to various concentrations of valproic acid. Specific data given are EC_10_ values (µM)

In a more differentiated approach, each effect expressed in ≥ 20% of all individuals was plotted separately for each compound, and EC_10_ values as well as lowest (LOECs) and no observed effect concentrations (NOECs) were determined (Table 4). Effects showing an expression profile of < 20% at the highest tested concentration only were interpreted as negative (−) and are not listed in Table 4. As an example, results for VPA are illustrated in Fig. 2. Specific data and graphs for the nine analogues of VPA are given as Figs. S1–S9 as well as Table S1 in the Supplemental Materials.

**Table 4:** Endpoint-specific effect concentrations (EC) as well as NOEC and LOEC of valproic acid in zebrafish (*Danio rerio*) embryos after 120 h of exposure

Effects	NOEC (µM)	LOEC (µM)	EC_10_ (µM)	EC_50_ (µM)
Coagulation	12.5	400	88	335
Blood congestion	100	200	119	n.d
Craniofacial deformation	50	100	52	142
Development retardation	200	400	n.d	n.d
Jitter/tremor	50	100	82	196
Lack of hatch	200	400	300	n.d
Pericardial edemata	25	50	38	90
Reduced heartbeat	200	400	209	n.d
Scoliosis/lordosis	6.25	50	59	230
Small eyes	200	400	n.d	n.d
Yolk edemata	12.5	25	82	n.d
Yolk sac absorption reduced	12.5	25	20	194

Effects showing an expression rate of < 20% at the highest test concentration only were interpreted as negative and are not included in this table. *n.d.* not determined (> highest test concentration)

**Fig. 3:**
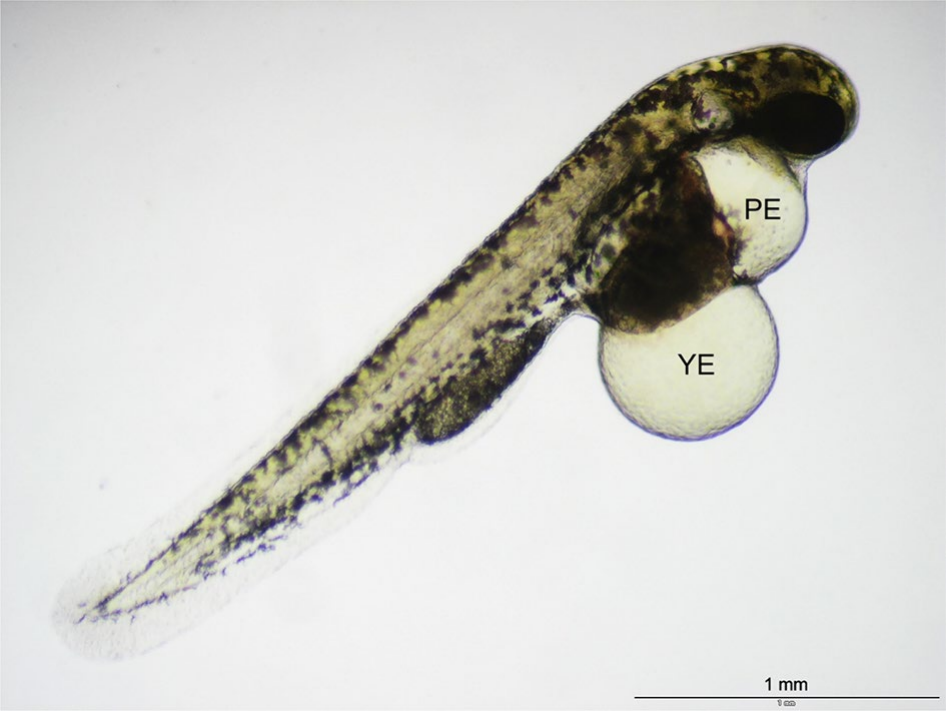
Pericardial edema (PE) and yolk edema (YE) as common effects recorded in zebrafish (*Danio rerio*) embryos after expsoure to VPA and all analogues (682 µM hexanoic acid; 96 hpf)

**Fig. 4:**
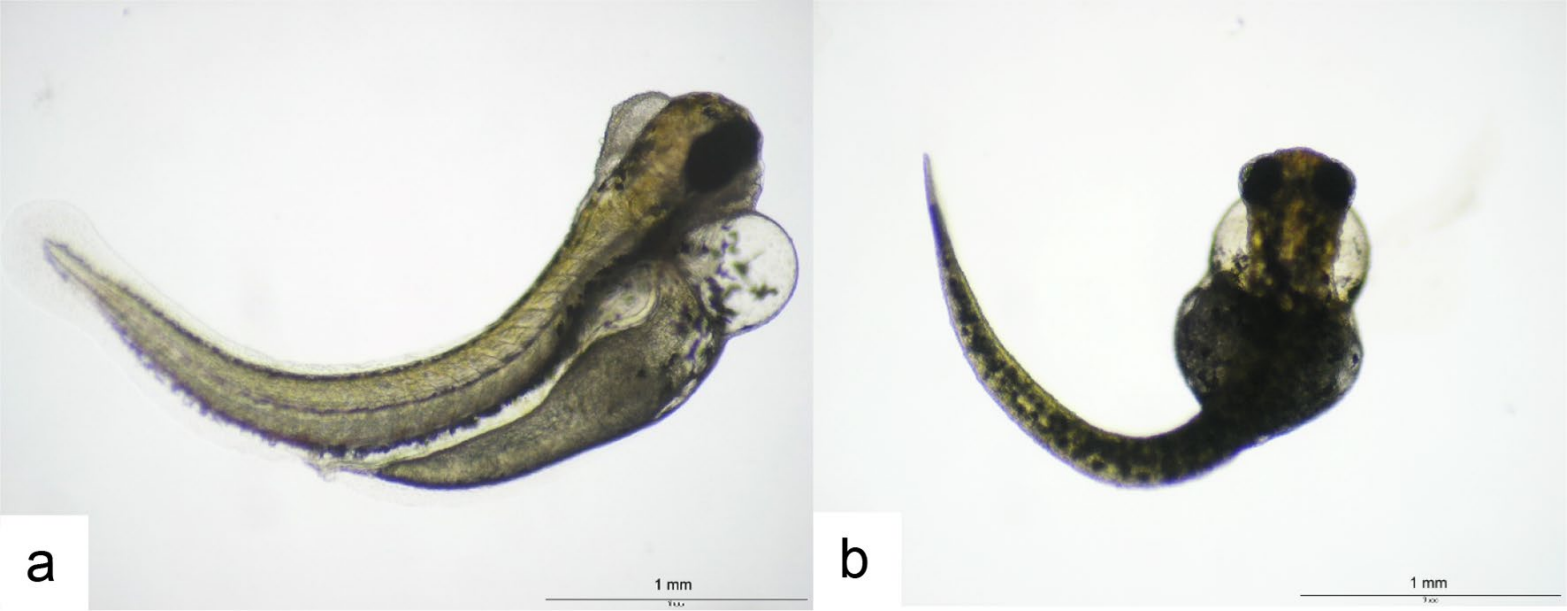
**a** Lordosis (2-methylhexanoic acid; 500 µM, 120 hpf) and **b** scoliosis (800 µM 2,2-dimethylvaleric acid; 120 hpf) as two of the most prominent observations after exposure of zebrafish (*Danio rerio*) embryos to VPA and its analogues

As common (unspecific) effects, pericardial and yolk edemata could be recorded with all substances even at the lowest test concentrations (Fig. 3). Most interestingly, with increasing exposure time, edemata proved reversible for all test substances even at concentrations up to their EC_50_ values. An in-depth analysis of effects, however, also identified pericardial edemata as the most sensitive endpoint for all substances except 2-methylhexanoic acid, while blood congestion and lack of otoliths were the second-most sensitive endpoints for 2-methylpentanoic acid, 4-pentenoic acid, hexanoic acid, 2,2-dimethylvaleric acid and 2-ethylbutyric acid. In contrast, for all compounds except 2-ethylbutyric acid, craniofacial deformation and scoliosis/lordosis, taking an intermediate position with respect to their EC_10_ data (Table S1), could be recorded as the most prominent observations (Fig. 4).

## Comparison of fish embryo test (FET) data with in vivo mouse data

**Fig. 5:**
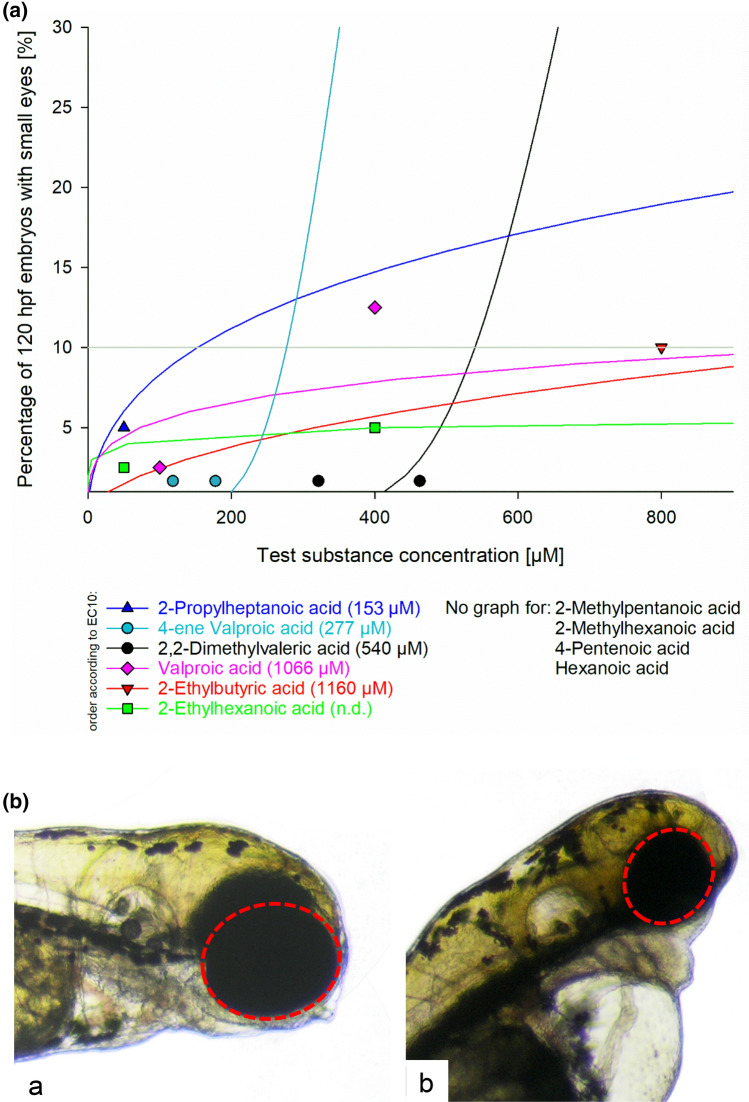
**a** Occurrence of the endpoint “small eyes” in zebrafish (*Danio rerio*) embryos exposed for 120 h to valproic acid and selected analogues. Data are given as % of affected embryos (*n* = 20–40). Due to the low occurrence of < 10% of effects at the highest test concentration and software limitations, the EC_10_ values computed for 2-propylheptanoic acid, valproic acid, 2-ethylbutyric acid and 2-ethylhexanoic acid were greater than the highest concentration tested in the FETs. Whereas embryos exposed to 2-propylheptanoic acid, 4-ene valproic acid, 2,2-dimethylvaleric acid, valproic acid, 2-etyhlbutyric acid and 2-ethylhexanoic acid showed an increase in the number of individuals with decreased eye size, embryos exposed to 2-methylpentanoic acid, 2-methylhexanoic acid, 4-pentenoic acid and hexanoic acid did not show an effect. **b** (a) Normal development of eyes in 120 h old zebrafish (*Danio rerio*) embryos. (b) Endpoint "small eyes" in 120 h old zebrafish embryos after treatment with 100 µM 2-*n*-propylheptanoic acid. Area of interest outlined in red

**Fig. 6:**
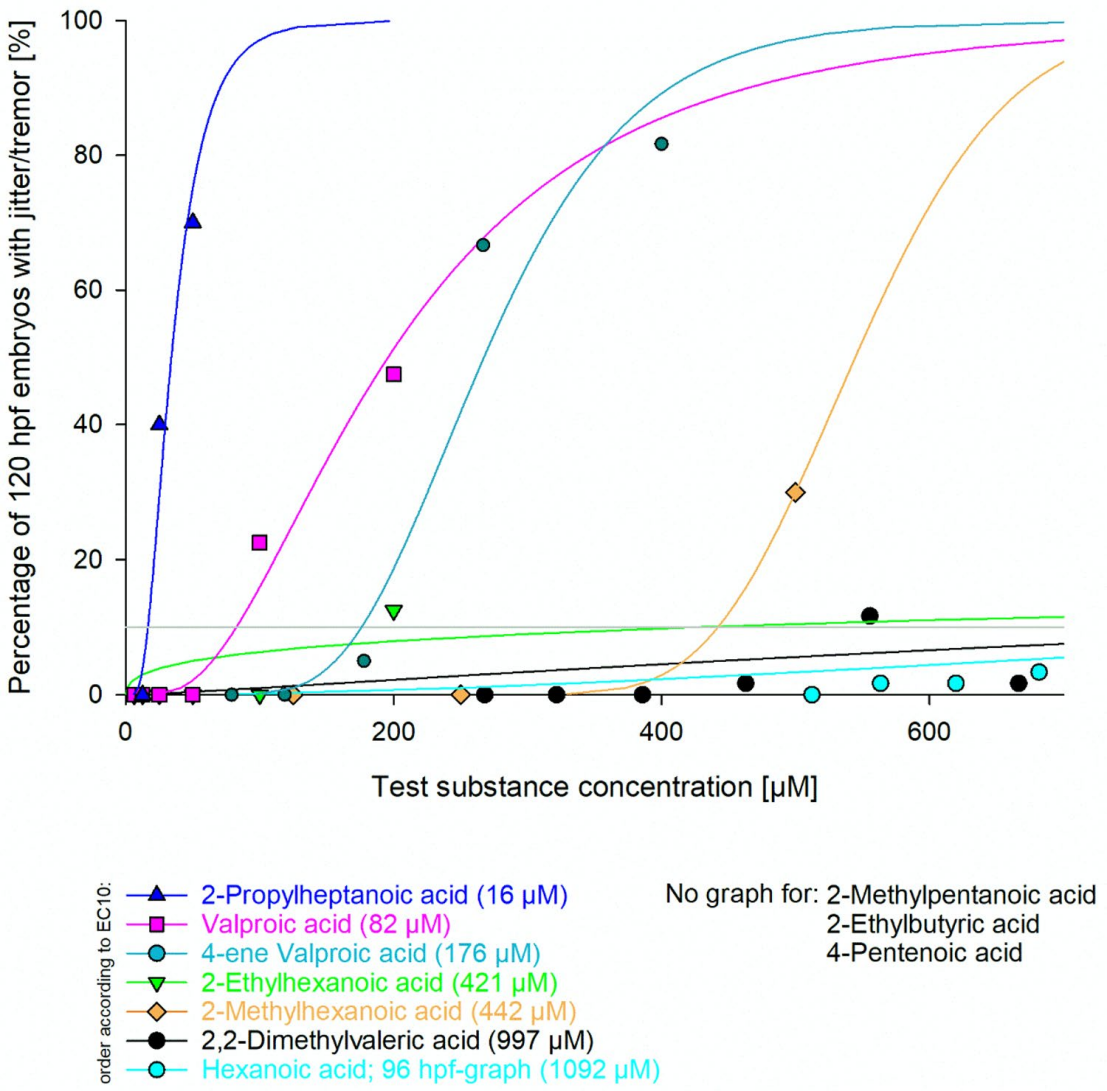
Occurrence of the endpoint “jitter/tremor” in zebrafish (*Danio rerio*) embryos exposed for 120 h to valproic acid and selected analogues. Data are given as % of affected embryos (*n* = 20–40). Whereas embryos exposed to 2-*n*-propylheptanoic acid, valproic acid, 4-ene valproic acid, 2-ethylhexanoic acid, 2-methylhexanoic acid, 2,2-dimethylvaleric acid and hexanoic acid showed an increase in the number of individuals with tremor, embryos exposed to 2-methylpentanoic acid, 2-ethylbutyric acid and 4-pentenoic acid did not show an effect

**Fig. 7:**
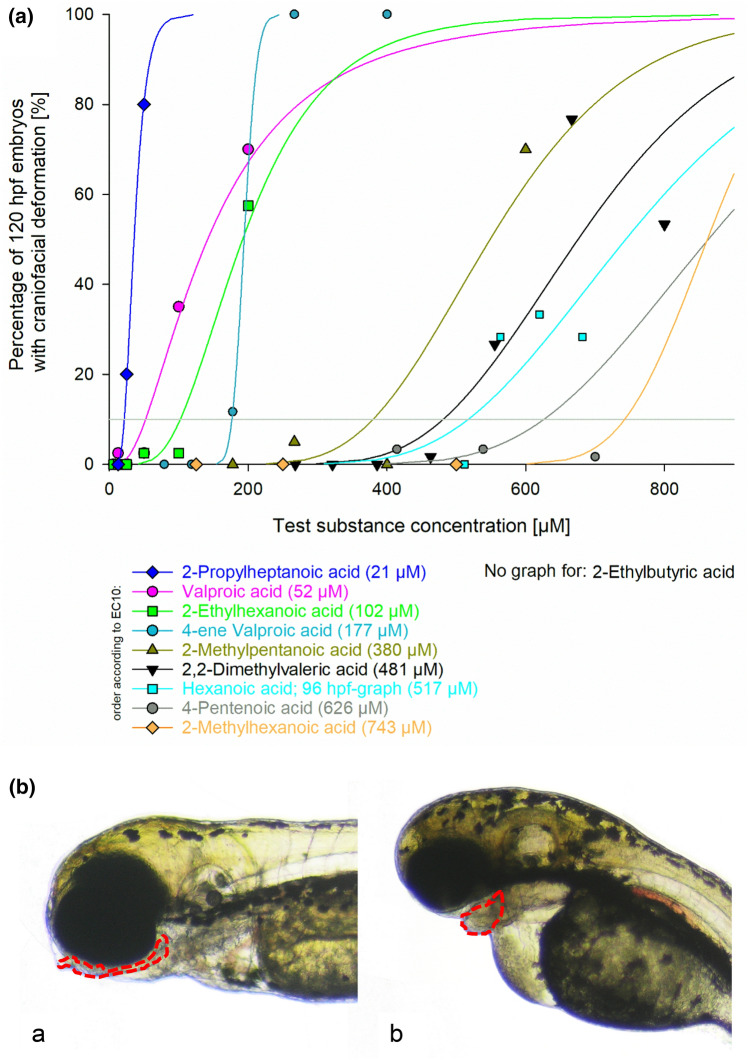
**a **Occurrence of the endpoint “craniofacial deformation” in zebrafish (*Danio rerio*) embryos exposed for 120 h to valproic acid and selected analogues. Data are given as % of affected embryos (*n* = 20–40). Whereas embryos exposed to 2-*n*-propylheptanoic acid, valproic acid, 2-ethylhexanoic acid, 4-ene valproic acid, 2-methylpentanoic acid, 2,2-dimethylvaleric acid, hexanoic acid, 4-pentenoic acid and 2-methylhexanoic acid showed an increase in the number of individuals with craniofacial deformation, embryos exposed to 2-ethylbutyric acid did not show an effect. **b** (a) Normal development of craniofacial structures in 120 h old zebrafish (*Danio rerio*) embryos. (b) Endpoint “craniofacial deformation” in zebrafish embryos after treatment with 400 µM valproic acid: lower jaws are massively reduced in extension. Area of interest outlined in red

For direct comparison with the in vivo neurotoxic potencies in mice, three specific effects were selected as potentially indicative of neurodevelopmental defects in zebrafish embryos: small eyes (Fig. 5b), jitter/tremor and craniofacial deformation (Fig. 7b). The occurrences of these three endpoints per compound are summarized in Figs. 5a, 6, 7a, which, however, only illustrate changes for those test substances that produced the effects.

Due to the low occurrence of < 10% of effects at the highest test concentration and software limitations, the EC_10_ values of some effects computed for 2-*n*-propylheptanoic acid, valproic acid, 2-ethylbutyric acid and 2-ethylhexanoic acid were greater than the highest concentration tested in the FETs. Sorting the compounds for these specific effects by EC_10_ values resulted in the following order starting with highest toxicity:

Small eyes:2-*n*-propylheptanoic acid > 4-*ene* valproic acid > 2,2-dimethylvaleric acid > valproic acid > 2-ethylbutyric acid > 2-ethylhexanoic acid.

Jitter/tremor:2-*n*-propylheptanoic acid > valproic acid > 4-*ene* valproic acid > 2-ethylhexanoic acid > 2-methylhexanoic acid > 2,2-dimethylvaleric acid > hexanoic acid.

Craniofacial deformation:2-*n*-propylheptanoic acid > valproic acid > 2-ethylhexanoic acid > 4-*ene* valproic acid > 2-methylpentanoic acid > 2,2-dimethylvaleric acid > hexanoic acid > 4-pentenoic acid > 2-methylhexanoic acid.

For the comparison between zebrafish embryo and mouse neurotoxicity, mouse potencies for exencephaly and zebrafish embryo data are summarized in Table 5. For reasons of simplification, the occurrence of an effect was marked either with an + (effect expressed) or − (effect not expressed). Since in some cases a clear categorization could not be established, specific comments were added as superscripts: Since for 2-ethylbutyric acid the endpoint “small eyes” was expressed in less than 20% of individuals, this effect was interpreted as negative ( −). In the cases of 2,2-dimethylvaleric acid and 2-methylhexanoic acid, the endpoint “jitter/tremor” could only be recorded after 96 h of exposure, since embryos exposed for 120 h were immobilized. In contrast, for hexanoic acid, the observation of “jitter/tremor” could be made in only 1 out of 3 replicates with an occurrence of > 50%. For 4-pentenoic acid and 2-methylhexanoic acid, “craniofacial deformation” was only evident at the highest test concentration; however, these embryos simultaneously also expressed several other severe effects (for details, see Table S1 in Supplemental Materials).

**Table 5:**
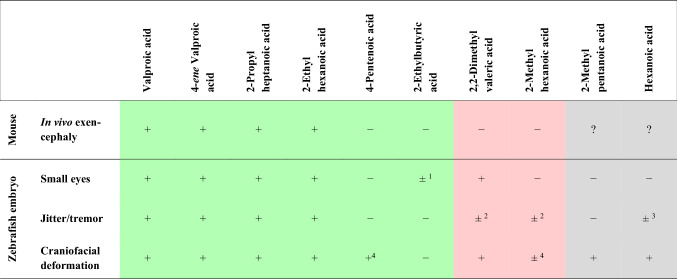
Comparison of in vivo mouse potencies for exencephaly and effects observed in zebrafish (*Danio rerio*) embryos

Fields shaded in green indicate similar trends in mouse and zebrafish; fields shaded in red indicate contradictory trends; grey fields indicate substances for which mouse data are lacking  + Effect expressed; − Effect not expressed

^1^Interpreted as negative ( −), since the number of embryos expressing this effect was < 20%. ^2^Effect observed in 96 h zebrafish embryos only (negative in 120 h embryos). ^3^In one replicate out of three only, 50% of zebrafish embryos expressed tremor. ^4^At highest concentration only

## Discussion

For the pre-screening of drugs suspected to be hazardous, the zebrafish embryo has repeatedly been advocated as a promising model (Beker van Woudenberg et al. 2014; Herrmann 1993; Kari et al. 2007; MacRae and Peterson 2015; Scholz et al. 2008; Yamashita et al. 2014; Yang et al. 2009). The present study had been designed to analyze the predictivity of observations obtained with embryos of a wild-type strain of the zebrafish (*Danio rerio*) for neurotoxic effects in mice after treatment with valproic acid (VPA) and nine selected analogues. In humans, application of VPA as an anticonvulsant drug increases the risk of neural tube defects by a factor of 10–20 (Spiegelstein et al. 2003). The phenotypes indicating neurotoxicity in mammals, however, are quite diverse: Whereas, e.g., VPA-induced neural tube defects in humans express themselves as *spina bifida* (Nanau and Neuman 2013), the reaction in mice is characterized by exencephaly (Nau et al. 1991; Nau and Löscher 1986). In rat medicated with VPA, neural tube effects could be recorded as “spina bifida occulta” with split vertebrae incapable of effectively protecting the neural tube (Duru and Ceylan 2019). According to Nau (1986), VPA causes neural tube defects in humans (spina bifida), mice and hamsters (exencephaly), but not in monkeys and rabbits. Finally, apart from zebrafish, mouse and rat, VPA has been tested both in vivo and in vitro in *Xenopus*, chicken, hamster, gerbil, rabbit, dog and rhesus monkeys; however, the most common effects were not neural tube defects, but skeletal defects in ribs, vertebrae, digits and craniofacial bones as manifested in ossification defects as well as abnormal numbers and shapes (Hill et al. 2010; Turgut et al. 2019).

In vertebrates, there are generally two alternatives to form the neural tube: (1) In primary neurulation, the cells surrounding the neural plate induce proliferation and invagination of neural plate cells, thus shaping the neural tube (Gilbert 2000; Yuskaitis and Pomeroy 2017). (2) In contrast, secondary neurulation is a process characterized by sinking of a solid cord of cells followed by subsequent formation of hollow neural tube. In fish, neurulation is exclusively secondary (Gilbert 2000; Yuskaitis and Pomeroy 2017), whereas in humans the process of neurulation can be subdivided into primary neurulation during weeks 3 and 4 of gestation and secondary neurulation between weeks 5 and 6 (Gilbert 2000; Greene and Copp 2014; Mitchell et al. 2004; Yuskaitis and Pomeroy 2017). In humans, secondary neurulation only starts, when primary neurulation has been completed and the posterior neuropore has been closed, with the latter also being the prime region of interest for spinal cord malformations such as *spina bifida* (Copp et al. 2015; Northrup and Volcik 2000; Yuskaitis and Pomeroy 2017).

Despite differential formation of neural tube in fish, open neural tubes have been documented in mutant zebrafish embryos that were deficient in, e.g., Nodal signaling (Aquilina-Beck et al. 2007; Kindt et al. 2018) or the cell adhesion protein *N*-adherin; (squint) ${sqt}^{cz35}$ (Yuskaitis and Pomeroy 2017) as well as ${sqt}^{cz35}$; ${cyc}^{294}$ (Feldman et al. 1998). Even Z*oep* mutants, usually expressing cyclops at high rates, did show the open neural tube phenotype (Ma et al. 2015). However, in all of these studies, whole mount in situ hybridization was necessary to make this effect detectable (e.g., Aquilina-Beck et al. 2007; Araya et al. 2016). Thus, due to differences in embryonic developmental processes between fish and humans, the classical deficits in neural tube closure such as open head or notochord or a complete lack of head formation (anencephaly; Kindt et al. 2018) cannot be observed in zebrafish embryos without specific markers (Lu et al. 2013; Ma et al. 2015).

In fact, using the simple four standard endpoints (coagulation, lack of somite formation, lack of heartbeat, lack of tail detachment) listed in the protocol for OECD TG 236 (OECD 2013), the present study also failed to identify deficits in neural tube closure after exposure to VPA and its analogues. However, inclusion of additional observations did allow the diagnosis of neurodevelopmental effects: (1) deformation of eyes (“small eyes”), (2) craniofacial deformation and (3) behavioral effects such as jitter/tremor. Deformation of eyes, namely the observation of “small eyes”, was selected as an endpoint for its immediate connection to the neural system of the embryo (Asharani et al. 2008; Bilotta et al. 2004; Kim et al. 2018; Roy et al. 2016; Santos-Ledo et al. 2011; Xin et al. 2015).

Craniofacial deformation was picked as an endpoint on the basis of established AOPs connecting histone deacetylase (HDAC) inhibition, a molecular initiating event (MIE), to defects in craniofacial formation (https://aopwiki.org/aops/274) (Kong et al. 2014; McGee-Lawrence and Westendorf 2011; Pillai et al. 2004; Rao and LaBonne 2018) and neural tube defects (https://aopwiki.org/aops/275) (Gurvich et al. 2005; Massa et al. 2005; Menegola et al. 2005; Murko et al. 2013) as an adverse outcome (AO). Since VPA has been shown to be an HDAC inhibitor in both mammals and zebrafish (Giavini and Menegola 2014; Gurvich et al. 2005; Li et al. 2016; Massa et al. 2005), this effect offers a valuable connection on a molecular base between the compounds investigated and morphological endpoints.

Jitter or tremor (Vaz et al. 2018) is defined as uncontrolled vigorous movement of the entire embryo without clear movement into one direction (Kalueff 2017; Kalueff et al. 2013; Santos et al. 2018). In more general terms, the manipulation of movement by environmental toxicants has recently received increasing attention as a quantitative marker of neurotoxicity (d'Amora and Giordani 2018; Legradi et al. 2015, 2018; Tierney 2011; Zindler et al. 2019a, b).

In search of surrogate endpoints in zebrafish embryos for neural tube effects, the combination of these three endpoints was analyzed to test the hypothesis by Beker van Woudenberg et al. (2014) that such a multi-endpoint approach would generally increase the sensitivity and predictivity of the FET for developmental (neuro)toxicity screening. Specifically, two basic questions formed the basis for the present study: (1) Would the accuracy of the zebrafish embryo as a model for the prediction of mammalian and human toxicity and teratogenicity benefit from an isolated assessment of all effects, if compared to a standard analysis combining all observations into one summary parameter (as stipulated in OECD TG 236)? (2) Which of the structurally similar VPA analogues would also be teratogenic in the zebrafish embryo, indicating that the zebrafish embryo would allow the prediction of known in vivo-negative and/or in vivo-positive potentials of VPA analogues?

With respect to the first (methodological) question, the comparison of isolated versus summary analysis of endpoints revealed that both approaches lead to similar conclusions as to the predictivity of effects in mammals (mice), however, with the isolated approach allowing a more straightforward comparison of FET and mice data. While a summarizing analysis of all effects followed by an alignment according to their EC_10_ values only allowed the identification of a *trend* (higher or lower toxicity), an isolated evaluation of more specific endpoints allowed a direct comparison of the frequencies of the three selected endpoints (eye development, craniofacial deformation and jitter/tremor) to mouse in vivo potencies. Furthermore, sorting the compounds for these specific effects by EC_10_ values resulted in an order similar to that found for general toxicity.

With respect to the second question (prediction of the teratogenic potentials), the results of the summary analysis revealed 2-*n*-propylheptanoic acid, valproic acid, 2-ethylhexanoic acid and 4-*ene* valproic acid as the most toxic compounds for fish embryos (cf. Fig. 1, Table 3) by aligning all ten substances tested according to their EC_10_ values. Given that all of these substances induced exencephaly in mice (Nau and Löscher 1986; Nau and Zierer 1982; Padmanabhan and Ahmed 1996; Paulson et al. 1985; Sonoda et al. 1990), results provide evidence of principal predictive power of the zebrafish embryo model.

The analysis of more specific endpoints thought to be suitable as surrogates of neural tube defects (tremor, craniofacial deformation, small eyes), however, not only correctly identified neural tube defect-positive analogues, but also correctly identified 4-pentenoic acid and 2-ethylbutyric acid as negative analogues (cf. Table 5). Only two compounds (2,2-dimethylvaleric acid and 2-methylhexanoic acid) were tested negative in the mouse model, but were predicted positive in the zebrafish embryo model, albeit evidence was not unequivocal in that tremor could not be observed consistently in all experimental groups and craniofacial deformation could only be detected at fairly high concentrations of 2-methylhexanoic acid. In fact, for all compounds craniofacial deformation took an intermediate position with respect to their EC_10_ data (cf. Table S1) except for 2-ethylbutyric acid.

For 2-methylpentanoic acid and hexanoic acid, a direct comparison was not possible due to a lack of information about in vivo potencies in the mouse model. However, based on the zebrafish embryo data, 2-methylpentanoic acid proved negative neurotoxic potency for “small eyes” and “jitter/tremor”, but positive for “craniofacial deformation”. Hexanoic acid would be regarded as negative for “small eyes”, but positive for “jitter/tremor” and “craniofacial deformation”.

Thus, for VPA and its analogues with known mammalian neurotoxic potency, a predictive power of about 75% could be concluded for the zebrafish embryo model. This rate is similar to the conclusions (≥ 80%) drawn from previous studies on the agreement of data obtained from zebrafish screening and data for mammalian developmental toxicity focusing on both morphological endpoints and/or gene expression (Bachmann 2002; Brannen et al. 2010; MacRae and Peterson 2015; Nagel 2002). Therefore, according to the evaluation guidelines of the European Centre for the Validation of Alternative Methods (ECVAM), the present study would score the zebrafish embryo as a “good” alternative toxicity assay (predictivity > 75%) (Genschow et al. 2002; Yang et al. 2009).

In fact, the predictivity of the zebrafish model could be further improved by additional modifications of the OECD TG 236 protocol. For substances affecting pH (like the acids tested in the present study), a more rigid adjustment of pH would be helpful. Although zebrafish embryos are fairly tolerant to pH variations between pH 6.5 and 8.5 (OECD 2013), it should be noted that pH may also profoundly affect the speciation and solubility of the test compounds by shifting the equilibrium of ionized to non-ionized molecules in the test solutions and, thus, changing the availability of the compounds to the zebrafish embryos. In case of pH adjustment, the overall range of EC_10_ values of the VPA analogues would have become broader due to differential absorption capacities, and the negative analogues would even have needed relatively higher (nominal) test concentrations for inducing effects than the positive analogues, thus confirming the current conclusions. For confirmation of this hypothesis, comparisons of the bioavailability for a pH adjusted versus non-adjusted test scenario are under investigation.

On the other hand, both approaches taken in the present study have limitations: The standard summary approach (one LC/EC value) was restricted to only provide a trend of general toxic activities of the substances, but inherently failed to make any prediction as to their in vivo potency. In contrast, due to considerable inter-individual variability especially with respect to specific effects, the isolated analysis of specific endpoints at concentrations < EC_10_ frequently suffered from a lack of a consistent dose–response relationship: The calculation of EC_10_ values inherently suffered from, e.g., (1) low percentages of affected embryos within all test concentrations, (2) the occurrence of effects solely at the highest test concentration or (3) ratios of < 10% individuals affected across all concentrations and replicates.

## Conclusions

The results of the present study clearly document that an extension of the number and an improved selection of endpoints make the zebrafish embryo toxicity test (FET; OECD 236) a promising basis for the development of a screening tool for the prediction of (neuro)developmental effects and teratogenicity in vertebrates. A differential assessment of selected specific endpoints can clearly improve the predictive power over that of the standardized summary approach using one parameter for the combination of all effects observed; depending on the observation, however, a clear distinction between + (in vivo positive) and – (in vivo negative) may become difficult due to inter-individual variability. In any case, however, the zebrafish embryo has the potential to bridge the gap between subcellular as well as cell-based in vitro systems and intact animal models. However, further research is required and should, e.g., include a well-based selection of effects related to the mammalian disease of interest and a well-justified guidance for the interpretation of effects occurring at low rates in only part of the experimental animals. Thus, identification and testing of new chemicals might be improved for immediate use for human society as, e.g., concluded by Gao et al. (2014) from their attempts to investigate the potential of zebrafish embryos to predict anti-cancer therapeutics.

## Electronic supplementary material

Below is the link to the electronic supplementary material.

Supplementary file1 (DOCX 9145 kb)
