# Supplementary material for: Neurotoxic effects in zebrafish embryos by valproic acid and nine of its analogues: the fish-mouse connection?
Source: Arch Toxicol. 2020 Oct 27;95(2):641–57. doi: 10.1007/s00204-020-02928-7 (PMC7870776; doi:10.1007/s00204-020-02928-7)
Supplement: Supplementary file 1 — Supplementary file1 (DOCX 9145 kb) [file 204_2020_2928_MOESM1_ESM.docx]

**Supplemental materials**

Table S1: No (NOECs) and lowest observed effect concentrations (LOECs) as well as EC_10_ concentrations for specific observations made in the fish embryo test (FET) with zebrafish (*Danio rerio*) embryos exposed to nine analogues of valproic acid. Most sensitive effects are listed in black fields; most prominent effects are listed in shaded fields. Data for valproic acid are given in Table 4.

|  | 2-Ethylhexanoic acid (µM) | | |  | 2-Propylheptanoic acid (µM) | | |  | 2-Methylpentanoic acid (µM) | | |
| --- | --- | --- | --- | --- | --- | --- | --- | --- | --- | --- | --- |
|  | NOEC | LOEC | EC_10_ |  | NOEC | LOEC | EC_10_ |  | NOEC | LOEC | EC_10_ |
| Coagulation | 6.25 | 50 | 109 |  | 12.5 | 25 | 44 |  | 600 | 900 | 615 |
| Blood congestion | ̶ | ̶ | ̶ |  | 12.5 | 50 | 25 |  | <177.7 | 600 | 742 |
| Body under-pigmentation | ̶ | ̶ | ̶ |  | ̶ | ̶ | ̶ |  | ̶ | ̶ | ̶ |
| Brain discoloration | ̶ | ̶ | ̶ |  | ̶ | ̶ | ̶ |  | ̶ | ̶ | ̶ |
| Chorda deformation | ̶ | ̶ | ̶ |  | ̶ | ̶ | ̶ |  | ̶ | ̶ | ̶ |
| Craniofacial deformation | 100 | 200 | 102 |  | 12.5 | 50 | 21 |  | 177.7 | 266.6 | 380 |
| Enlarged otic vesicles | ̶ | ̶ | ̶ |  | ̶ | ̶ | ̶ |  | ̶ | ̶ | ̶ |
| Eye under-pigmentation | ̶ | ̶ | ̶ |  | ̶ | ̶ | ̶ |  | ̶ | ̶ | ̶ |
| Head deformation | ̶ | ̶ | ̶ |  | 25 | 50 | 29 |  | ̶ | ̶ | ̶ |
| Jitter/tremor | 100 | 200 | 421 |  | <16 | 50 | 16 |  | ̶ | ̶ | ̶ |
| Lack of hatch | 50 | 400 | 91 |  | 12.5 | 25 | 44 |  | 600 | 900 | 615 |
| Lack of heartbeat | 200 | 400 | 318 |  | ̶ | ̶ | ̶ |  | ̶ | ̶ | ̶ |
| Lack of movement | ̶ | ̶ | ̶ |  | ̶ | ̶ | ̶ |  | ̶ | ̶ | ̶ |
| Lack of otoliths | ̶ | ̶ | ̶ |  | ̶ | ̶ | ̶ |  | ̶ | ̶ | ̶ |
| Loss of somite differentiation | ̶ | ̶ | ̶ |  | ̶ | ̶ | ̶ |  | ̶ | ̶ | ̶ |
| Lying in lateral position | ̶ | ̶ | ̶ |  | ̶ | ̶ | ̶ |  | ̶ | ̶ | ̶ |
| Pectoral fins deformed | ̶ | ̶ | ̶ |  | ̶ | ̶ | ̶ |  | ̶ | ̶ | ̶ |
| Pericardial edemata | 25 | 50 | 65 |  | <12.5 | 12.5 | 11 |  | 266.6 | 600 | 373 |
| Reduced heartbeat | 100 | 200 | n.d. |  | 25 | 50 | 39 |  | ̶ | ̶ | ̶ |
| Reduced otic vesicles | ̶ | ̶ | ̶ |  | ̶ | ̶ | ̶ |  | ̶ | ̶ | ̶ |
| Scoliosis/lordosis | 12.5 | 200 | 651 |  | 25 | 50 | 41 |  | 266.6 | 600 | 393 |
| Small eyes | 25 | 400 | n.d. |  | 25 | 50 | 153 |  | ̶ | ̶ | ̶ |
| Yolk discoloration | ̶ | ̶ | ̶ |  | ̶ | ̶ | ̶ |  | ̶ | ̶ | ̶ |
| Yolk edemata | 50 | 100 | 105 |  | <10 | 50 | 10 |  | 400 | 600 | 552 |
| Yolk sac absorption reduced | ̶ | ̶ | ̶ |  | ̶ | ̶ | ̶ |  | ̶ | ̶ | ̶ |
| Yolk sac deformation | ̶ | ̶ | ̶ |  | ̶ | ̶ | ̶ |  | ̶ | ̶ | ̶ |

Note: Pericardial and yolk edemata appeared with all substances tested (even at the lowest test concentrations). Edemata formation may be reversible over time.

Table S1 (continued): No (NOECs) and lowest observed effect concentrations (LOECs) as well as EC_10_ concentrations for specific observation made in the fish embryo test (FET) with zebrafish (*Danio rerio*) embryos exposed to nine analogues of valproic acid. Most sensitive effects are listed in black fields; most prominent effects are listed in shaded fields.

|  | 2-Methylhexanoic acid (µM) | | |  | 2-Ethylbutyric acid (µM) | | |  | 2,2-Dimethylvaleric acid (µM) | | |
| --- | --- | --- | --- | --- | --- | --- | --- | --- | --- | --- | --- |
|  | NOEC | LOEC | EC_10_ |  | NOEC | LOEC | EC_10_ |  | NOEC | LOEC | EC_10_ |
| Coagulation | 500 | 1000 | 519 |  | 400 | 800 | 529 |  | 666.6 | 800 | 701 |
| Blood congestion | 250 | 500 | 459 |  | 400 | 800 | 700 |  | 462.9 | 555.5 | 604 |
| Body under-pigmentation | ̶ | ̶ | ̶ |  | ̶ | ̶ | ̶ |  | ̶ | ̶ | ̶ |
| Brain discoloration | ̶ | ̶ | ̶ |  | ̶ | ̶ | ̶ |  | 555.5 | 666.6 | 637 |
| Chorda deformation | ̶ | ̶ | ̶ |  | ̶ | ̶ | ̶ |  | 666.6 | 800 | 750 |
| Craniofacial deformation | 500 | 1000 | 743 |  | ̶ | ̶ | ̶ |  | 462.9 | 555.5 | 481 |
| Enlarged otic vesicles | ̶ | ̶ | ̶ |  | ̶ | ̶ | ̶ |  | ̶ | ̶ | ̶ |
| Eye under-pigmentation | ̶ | ̶ | ̶ |  | ̶ | ̶ | ̶ |  | ̶ | ̶ | ̶ |
| Head deformation | ̶ | ̶ | ̶ |  | ≤800 | 800 | 1160 |  | 555.5 | 666.6 | 637 |
| Jitter/tremor | <442 | 500 | 442 |  | ̶ | ̶ | ̶ |  | ≤666.6 | 666.6 | 977 |
| Lack of hatch | ̶ | ̶ | ̶ |  | 400 | 800 | 525 |  | 555.5 | 666.6 | 627 |
| Lack of heartbeat | ̶ | ̶ | ̶ |  | 400 | 800 | 622 |  | 666.6 | 800 | 764 |
| Lack of movement | ̶ | ̶ | ̶ |  | ̶ | ̶ | ̶ |  | 462.9 | 555.5 | 501 |
| Lack of otoliths | ̶ | ̶ | ̶ |  | values non-calculable | | |  | 666.6 | 800 | 772 |
| Loss of somite differentiation | ̶ | ̶ | ̶ |  | ̶ | ̶ | ̶ |  | 385.8 | 462.9 | 520 |
| Lying in lateral position | ̶ | ̶ | ̶ |  | ̶ | ̶ | ̶ |  | 385.8 | 800 | 422 |
| Pectoral fins deformed | ̶ | ̶ | ̶ |  | ̶ | ̶ | ̶ |  | 555.5 | 666.6 | 599 |
| Pericardial edemata | 250 | 500 | 385 |  | 200 | 400 | 363 |  | 385.8 | 462.9 | 426 |
| Reduced heartbeat | ̶ | ̶ | ̶ |  | ≤800 | 800 | n.d. |  | 555.5 | 666.6 | 679 |
| Reduced otic vesicles | ̶ | ̶ | ̶ |  | ̶ | ̶ | ̶ |  | 666.6 | 800 | 759 |
| Scoliosis/lordosis | 250 | 500 | 383 |  | 800 | >800 | 892 |  | 385.8 | 462.9 | 663 |
| Small eyes | ̶ | ̶ | ̶ |  | 400 | 800 | 1160 |  | 462.9 | 666.6 | 540 |
| Yolk discoloration | ̶ | ̶ | ̶ |  | ̶ | ̶ | ̶ |  | 462.9 | 555.5 | 469 |
| Yolk edemata | 250 | 500 | 402 |  | ̶ | ̶ | ̶ |  | 462.9 | 800 | 535 |
| Yolk sac absorption reduced | ̶ | ̶ | ̶ |  | ̶ | ̶ | ̶ |  | 462.9 | 555.5 | 502 |
| Yolk sac deformation | ̶ | ̶ | ̶ |  | ̶ | ̶ | ̶ |  | ̶ | ̶ | ̶ |

Note: Pericardial and yolk edemata appeared with all substances tested (even at the lowest test concentrations). Edemata formation may be reversible over time.Table S1 (continued): No (NOECs) and lowest observed effect concentrations (LOECs) as well as EC_10_ concentrations for specific observation made in the fish embryo test (FET) with zebrafish (*Danio rerio*) embryos exposed to nine analogues of valproic acid Most sensitive effects are listed in black fields; most prominent effects are listed in shaded fields.

|  | 4-Pentenoic acid (µM) | | |  | 4-*ene* Valproic acid (µM) | | |  | Hexanoic acid (µM) | | |
| --- | --- | --- | --- | --- | --- | --- | --- | --- | --- | --- | --- |
|  | NOEC | LOEC | EC_10_ |  | NOEC | LOEC | EC_10_ |  | NOEC | LOEC | EC_10_ |
| Coagulation | 538.66 | 910.33 | 697 |  | 400 | >400 | 328 |  | 512.26 | 563.49 | 557 |
| Blood congestion | <414.35 | 414.35 | 654 |  | 118.5 | 177.7 | 154 |  | 512.26 | 563.49 | 510 |
| Body under-pigmentation | ̶ | ̶ | ̶ |  | 400 | >400 | 468 |  | ̶ | ̶ | ̶ |
| Brain discoloration | ̶ | ̶ | ̶ |  | ̶ | ̶ | ̶ |  | ̶ | ̶ | ̶ |
| Chorda deformation | ̶ | ̶ | ̶ |  | ̶ | ̶ | ̶ |  | ̶ | ̶ | ̶ |
| Craniofacial deformation | <414.35 | 414.35 | 626 |  | 118.5 | 177.7 | 177 |  | 512.26 | 563.49 | 517 |
| Enlarged otic vesicles | ̶ | ̶̶ | ̶ |  | 400 | >400 | 525 |  | ̶ | ̶ | ̶ |
| Eye under-pigmentation | ̶ | ̶ | ̶ |  | 400 | >400 | 329 |  | ̶ | ̶ | ̶ |
| Head deformation | ̶ | ̶ | ̶ |  | ̶ | ̶ | ̶ |  | ̶ | ̶ | ̶ |
| Jitter/tremor | ̶ | ̶ | ̶ |  | 118.5 | <118.5 | 176 |  | >681.81 | 681.81 | 1092 (96hpf) |
| Lack of hatch | 700.26 | 910.33 | 875 |  | ̶ | ̶ | ̶ |  | ̶ | ̶ | ̶ |
| Lack of heartbeat | ̶ | ̶ | ̶ |  | 400 | >400 | 468 |  | 619.83 | 681.81 | 897 |
| Lack of movement | 700 .26 | 910.33 | 822 |  | 266.6 | >266.6 | 395 |  | >456 | 563.49 | 456 |
| Lack of otoliths | ̶ | ̶ | ̶ |  | ̶ | ̶ | ̶ |  | ̶ | ̶ | ̶ |
| Loss of somite differentiation | 538.66 | 700.26 | 645 |  | 266.6 | >266.6 | 322 |  | >460 | 750 | 460 |
| Lying in lateral position | 700.26 | 910.33 | 770 |  | 177.7 | >177.7 | 189 |  | >453 | 563.49 | 453 |
| Pectoral fins deformed | ̶ | ̶ | ̶ |  | >177.7 | 177.7 | 234 |  | ̶ | ̶ | ̶ |
| Pericardial edemata | 538.66 | 700.26 | 568 |  | 79.01 | >79.01 | 93 |  | values non-calculable | | |
| Reduced heartbeat | 700.26 | 910.33 | 854 |  | 266.6 | 400 | 429 |  | 563.49 | 750 | 848 |
| Reduced otic vesicles | ̶ | ̶ | ̶ |  | 400 | >400 | 520 |  | 681.81 | 750 | n.d. |
| Scoliosis/lordosis | 700.26 | 910.33 | 747 |  | 266.6 | >266.6 | 289 |  | >490 | 563.49 | 490 |
| Small eyes | ̶ | ̶ | ̶ |  | 266.6 | >266.6 | 277 |  | ̶ | ̶ | ̶ |
| Yolk discoloration | 538.66 | 700.26 | 596 |  | 79.01 | 177.7 | 233 |  | >191 | 512.26 | 191 |
| Yolk edemata | 538.66 | 700.26 | 618 |  | 177.7 | >177.7 | 245 |  | >412 | 512.26 | 412 |
| Yolk sac absorption reduced | ̶ | ̶ | ̶ |  | 118.5 | >118.5 | 166 |  | >512.26 | 512.26 | 466 |
| Yolk sac deformation | ̶ | ̶ | ̶ |  | 400 | >400 | 413 |  | ̶ | ̶ | ̶ |

Note: Pericardial and yolk edemata appeared with all substances tested (even at the lowest test concentrations). Edemata formation may be reversible over time.

**Dose-response curves for specific effects following exposure of zebrafish (*Danio rerio*) embryos to 9 selected valproic acid analogues**


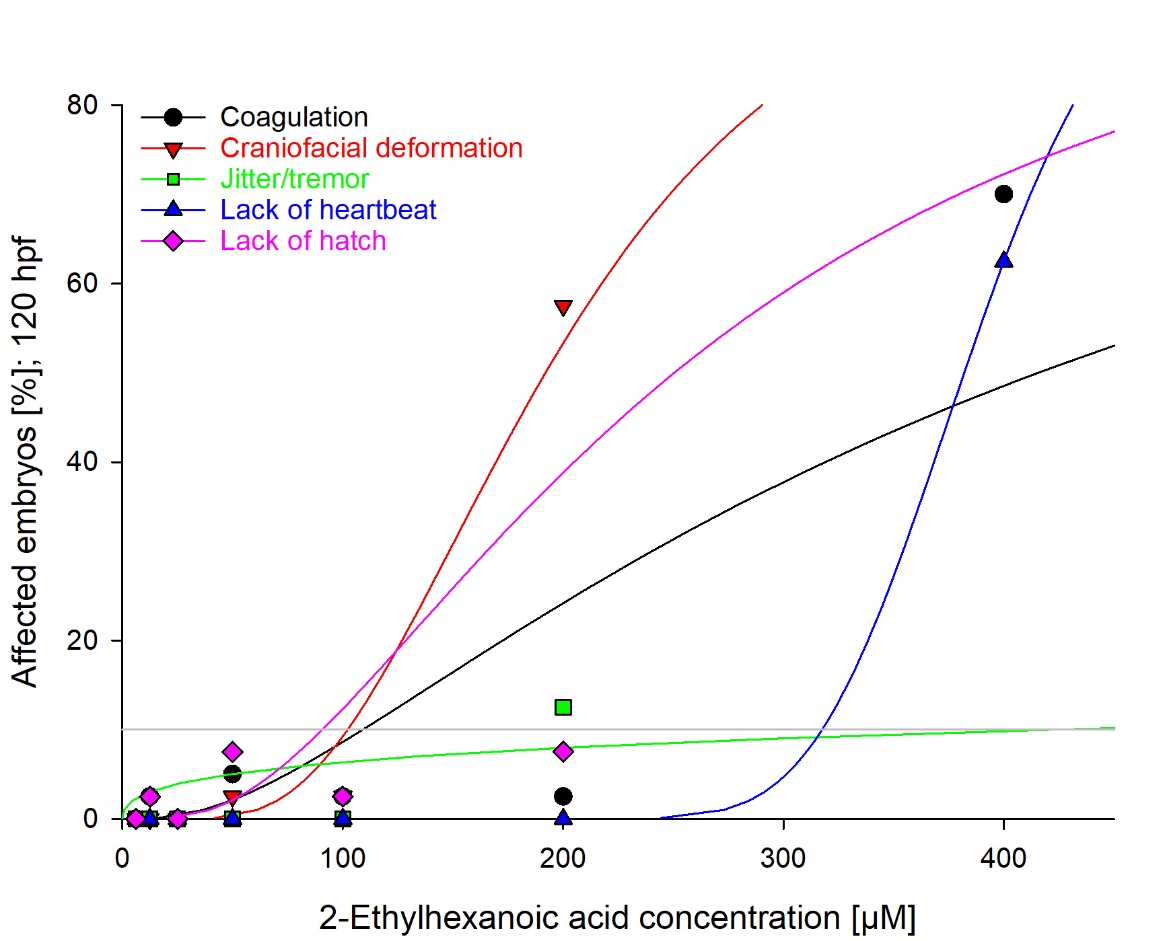


EC_10_ (µM)

109

102

421

318

91


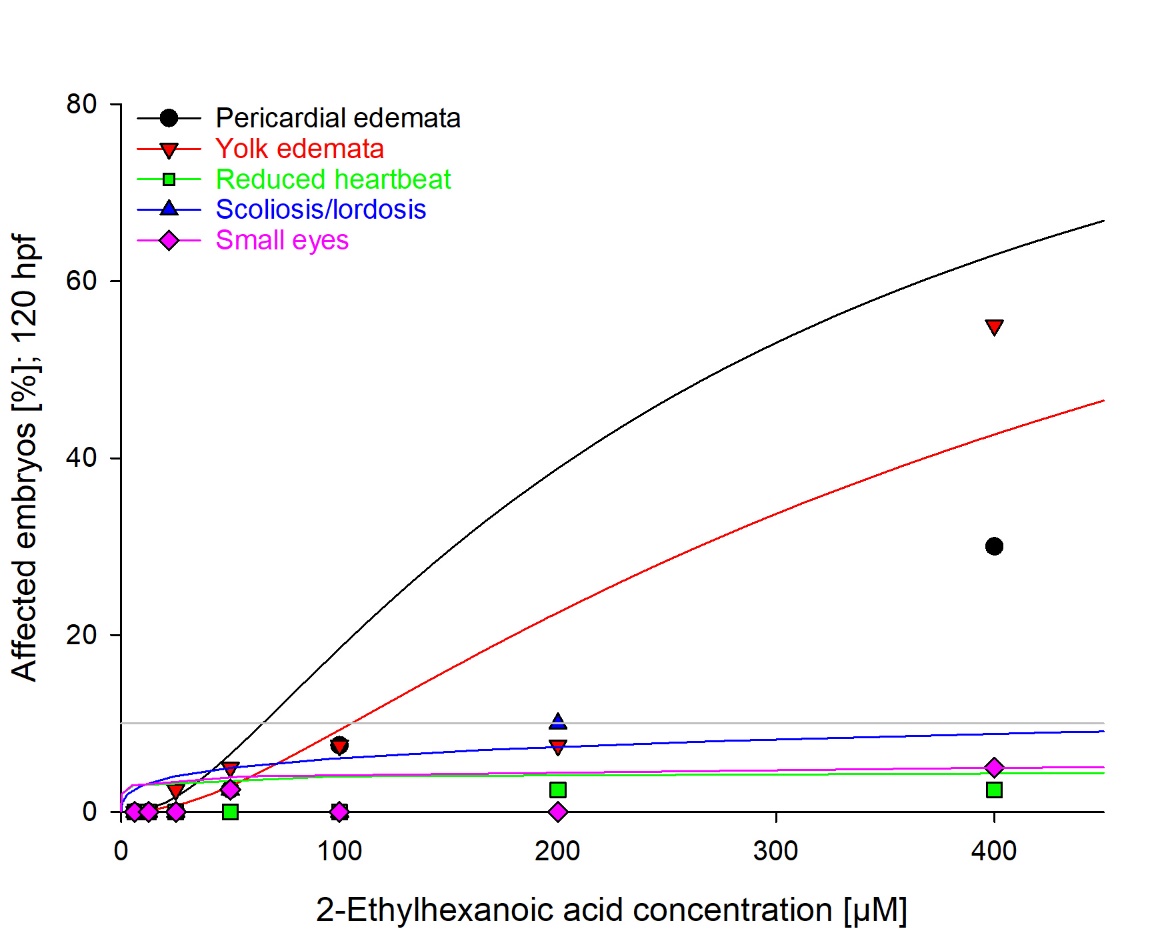


EC_10_ (µM)

65

105

n.d.

651

n.d.

Fig. S1: Dose-response curves for specific effects induced by exposure of zebrafish (*Danio rerio*) embryos to 2-ethylhexanoic acid for 120 h. Data are given as percentage of individuals affected (%) in n = 20 - 40 replicates.


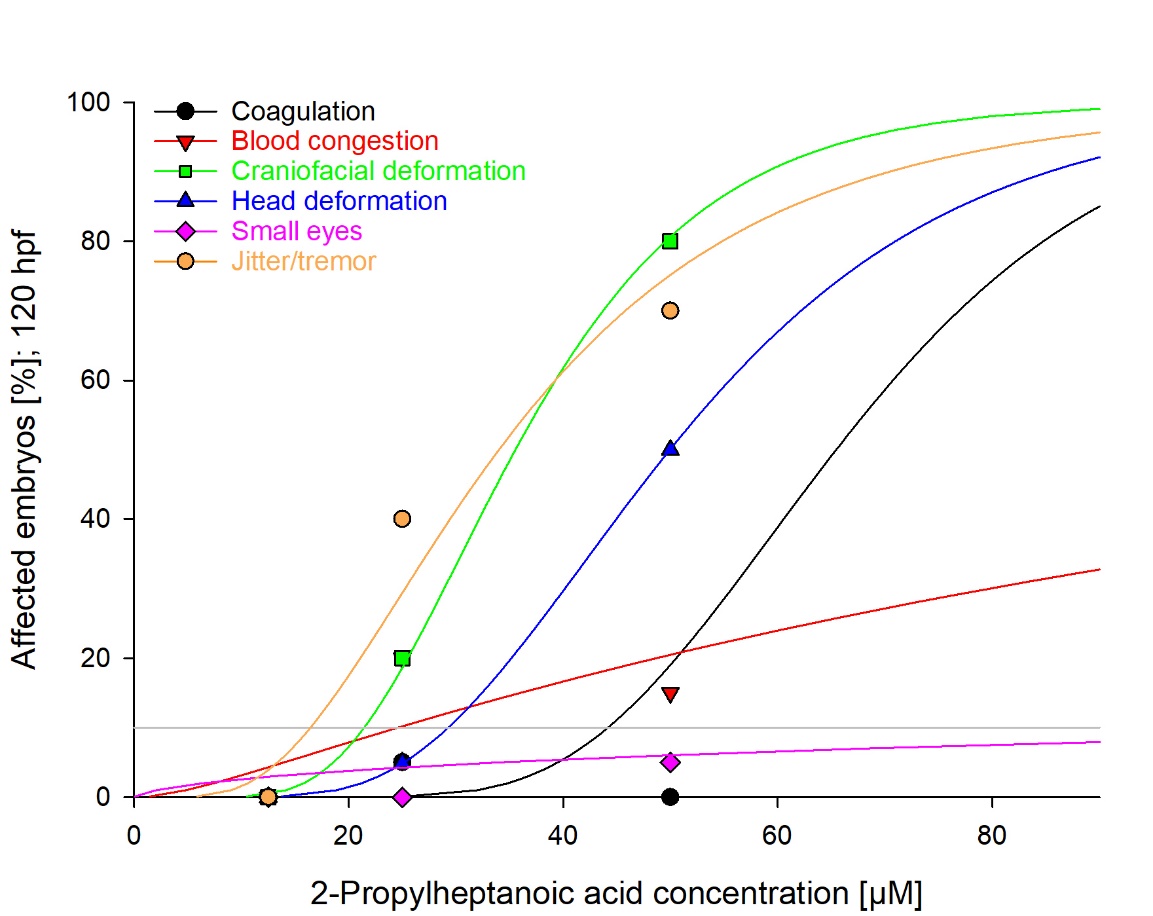


EC_10_ (µM)

44

25

21

29

153

16


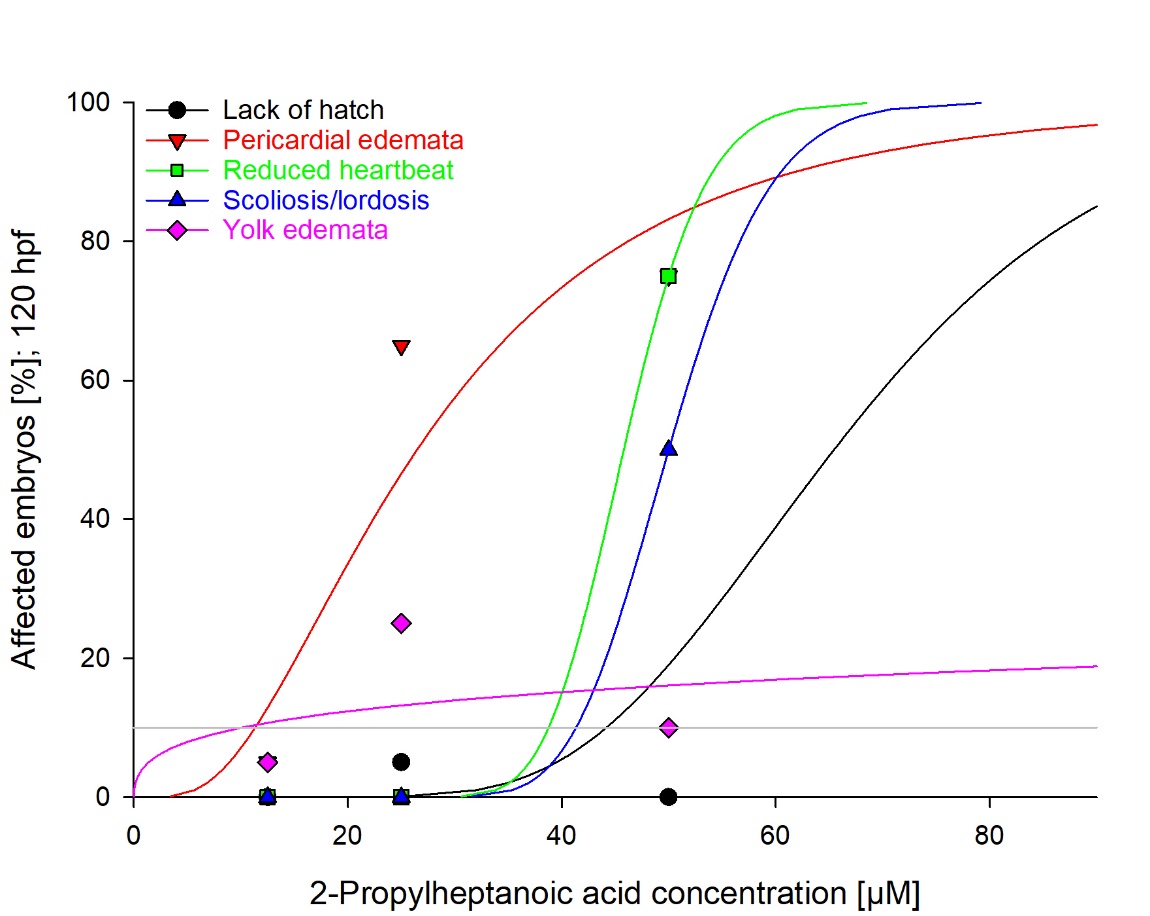


EC_10_ (µM)

44

11

39

41

10

Fig. S2: Dose-response curves for specific effects induced by exposure of zebrafish (*Danio rerio*) embryos to 2-propylheptanoic acid for 120 h. Data are given as percentage of individuals affected (%) in n = 20 - 40 replicates.


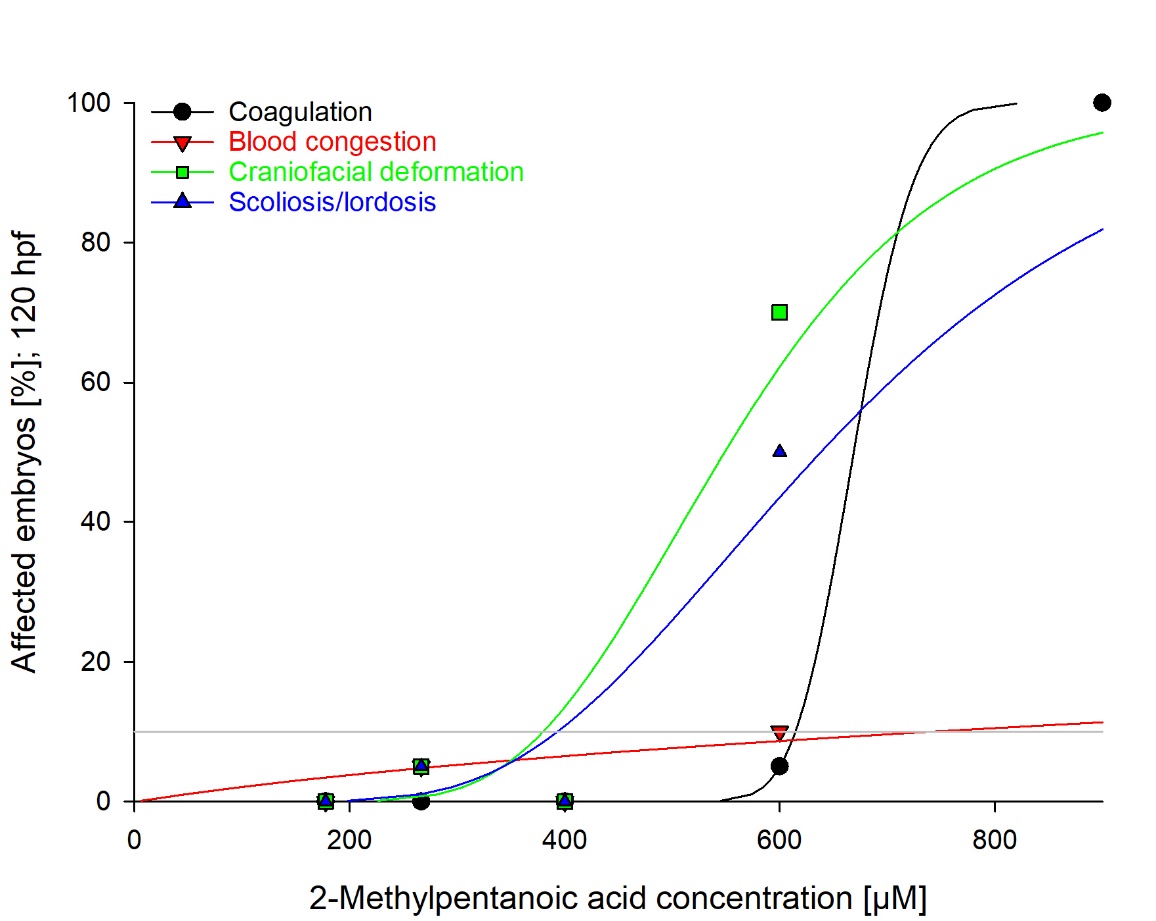


EC_10_ (µM)

615

742

380

393


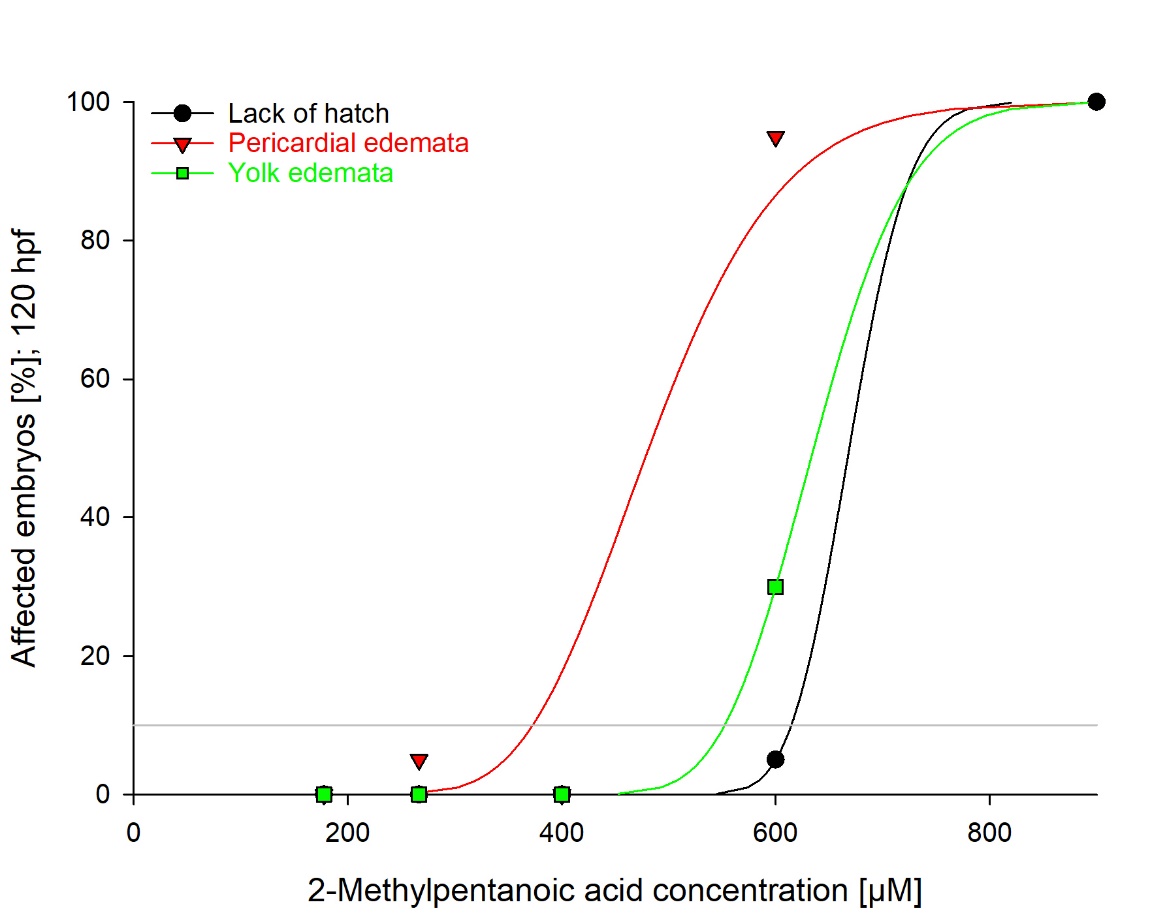


EC_10_ (µM)

615

373

552

Fig. S3: Dose-response curves for specific effects induced by exposure of zebrafish (*Danio rerio*) embryos to 2-methylpentanoic acid for 120 h. Data are given as percentage of individuals affected (%) in n = 20 - 40 replicates.


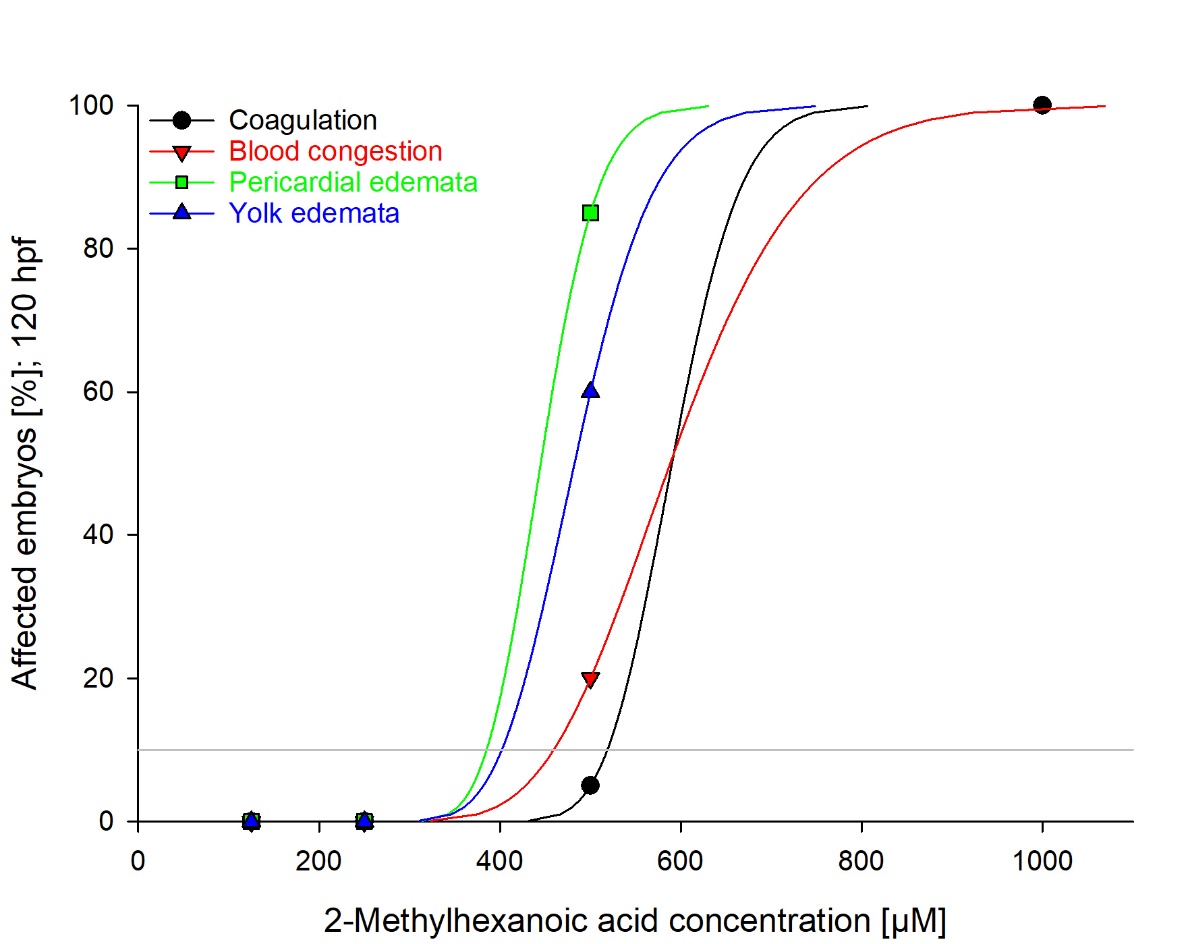


EC_10_ (µM)

519

459

385

402


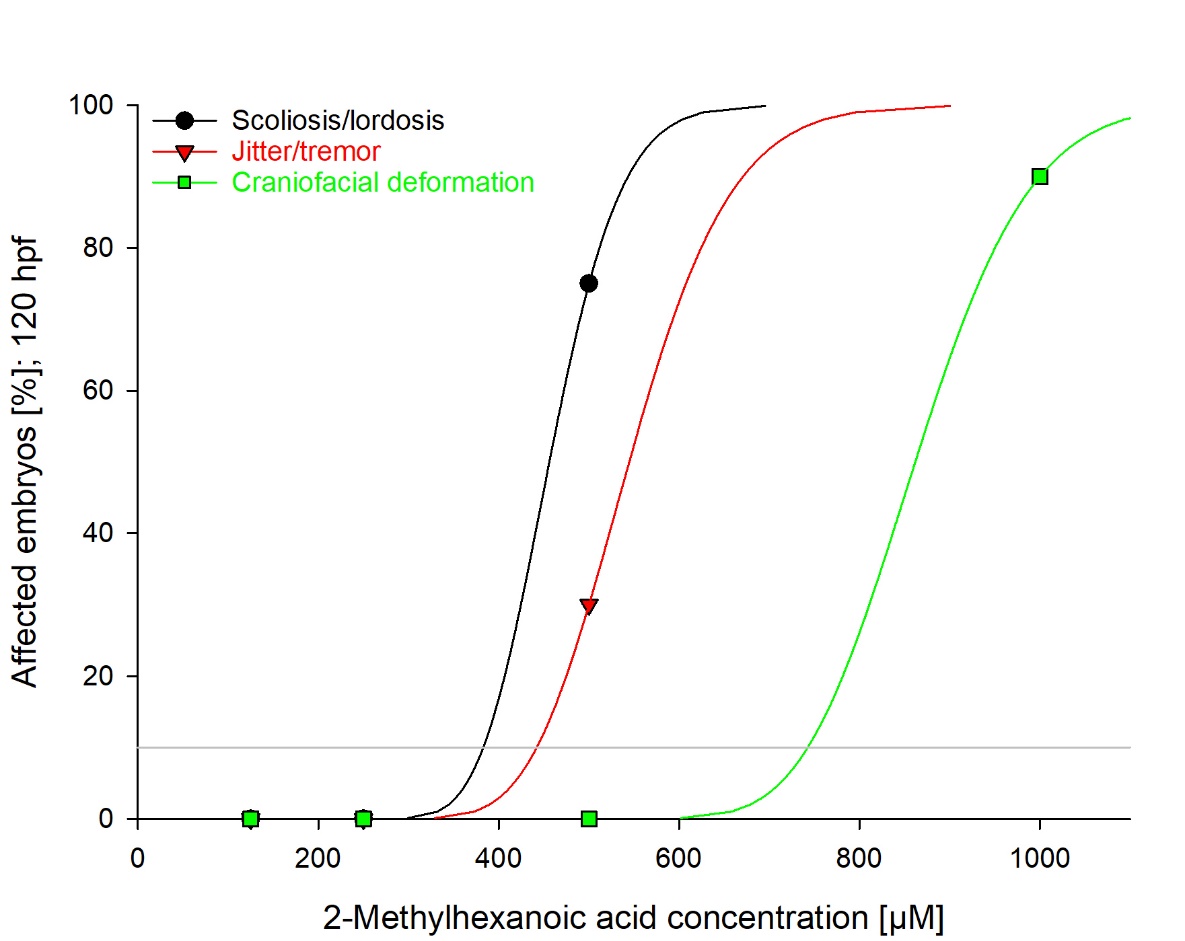


EC_10_ (µM)

383

442

743

Fig. S4: Dose-response curves for specific effects induced by exposure of zebrafish (*Danio rerio*) embryos to 2-methylhexanoic acid for 120 h. Data are given as percentage of individuals affected (%) in n = 20 - 40 replicates.


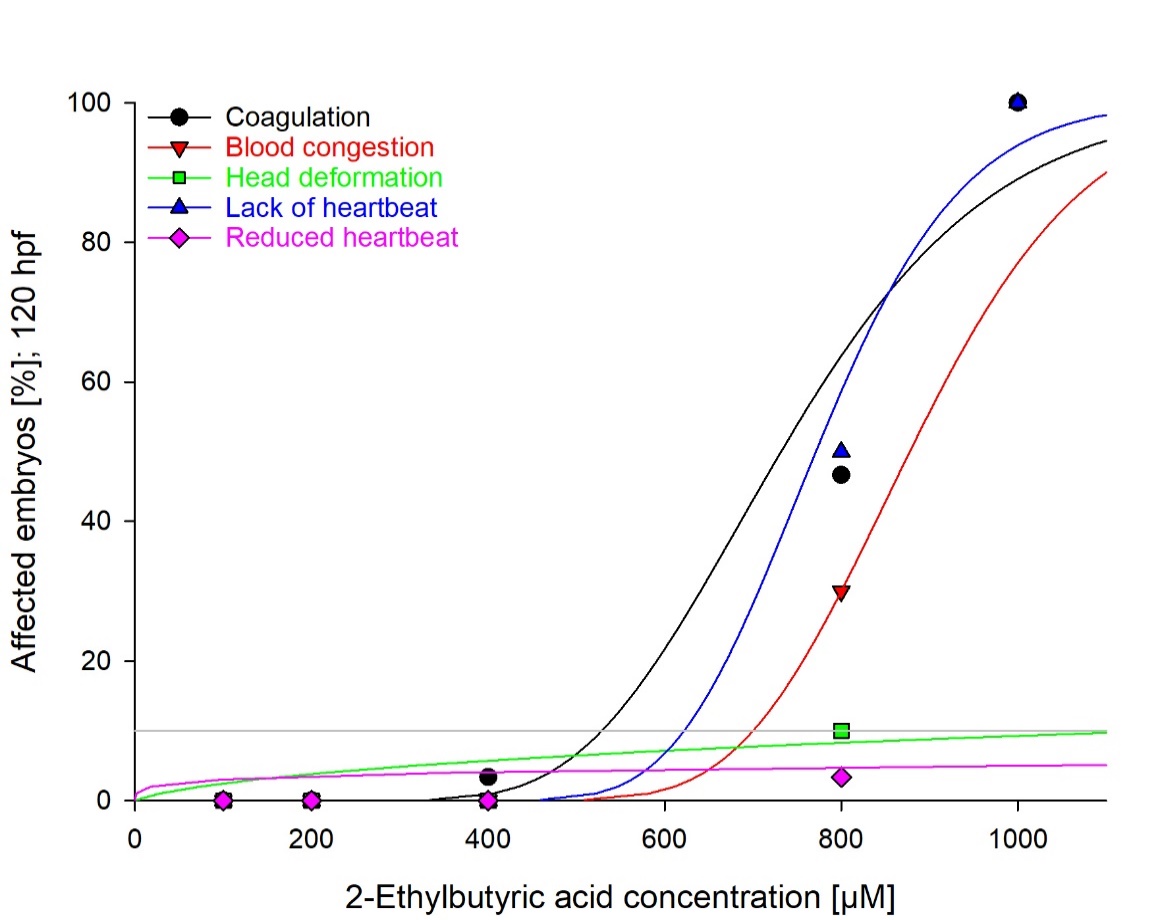


EC_10_ (µM)

529

700

1160

622

n.d.


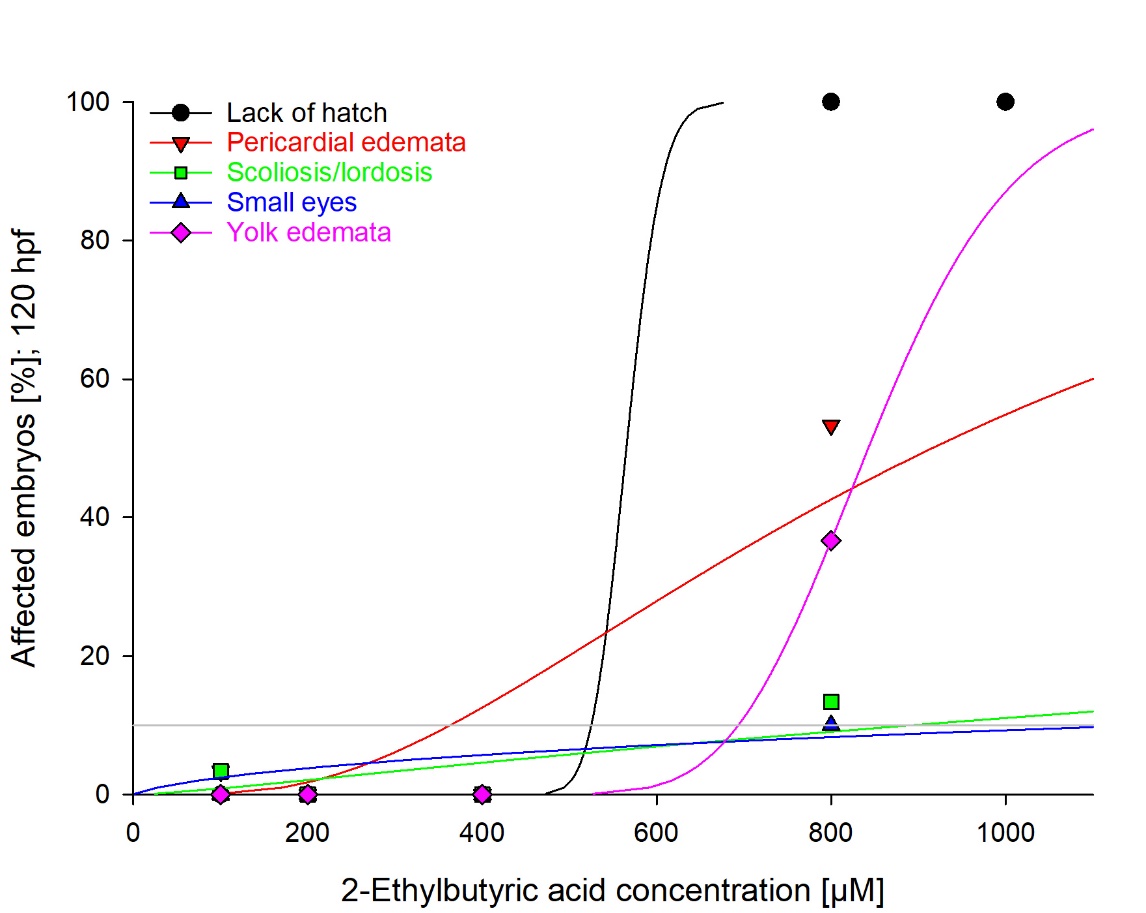


EC_10_ (µM)

525

363

892

1160

693

Fig. S5: Dose-response curves for specific effects induced by exposure of zebrafish (*Danio rerio*) embryos to 2-ethylbutyric acid for 120 h. Data are given as percentage of individuals affected (%) in n = 20 - 40 replicates.


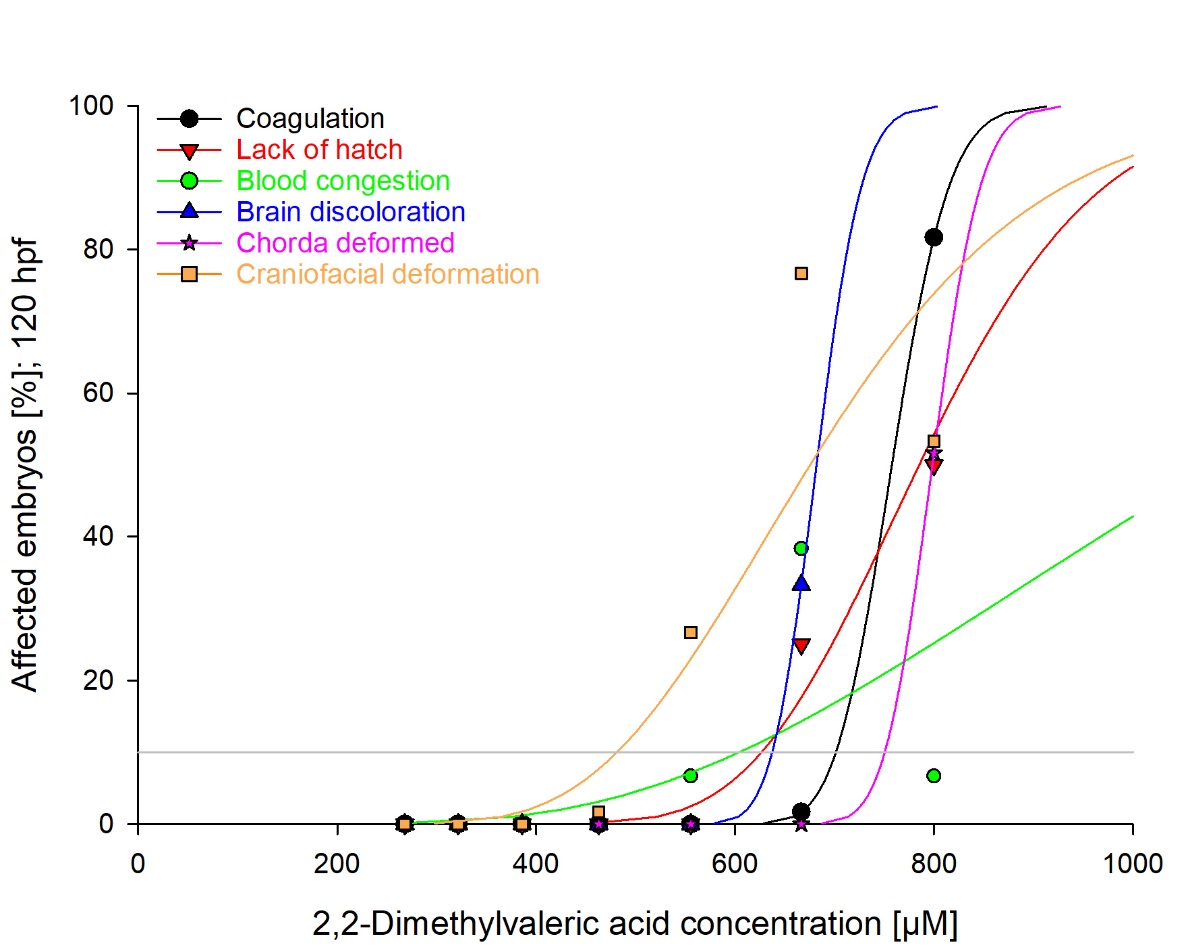


EC_10_ (µM)

701

627

604

637

750

481


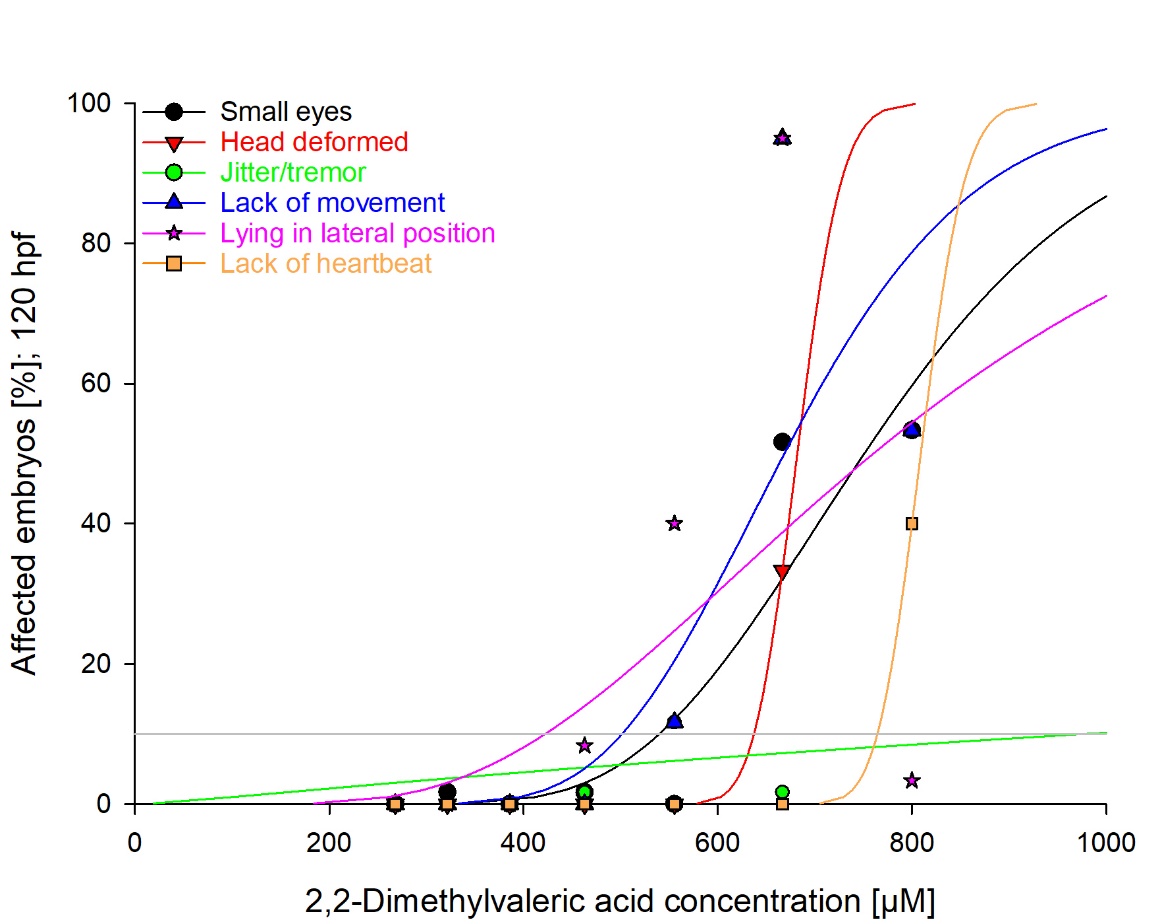


EC_10_ (µM)

540

637

977

501

422

764

Fig. S6: Dose-response curves for specific effects induced by exposure of zebrafish (*Danio rerio*) embryos to 2,2-dimethylvaleric acid for 120 h. Data are given as percentage of individuals affected (%) in n = 20 - 40 replicates.


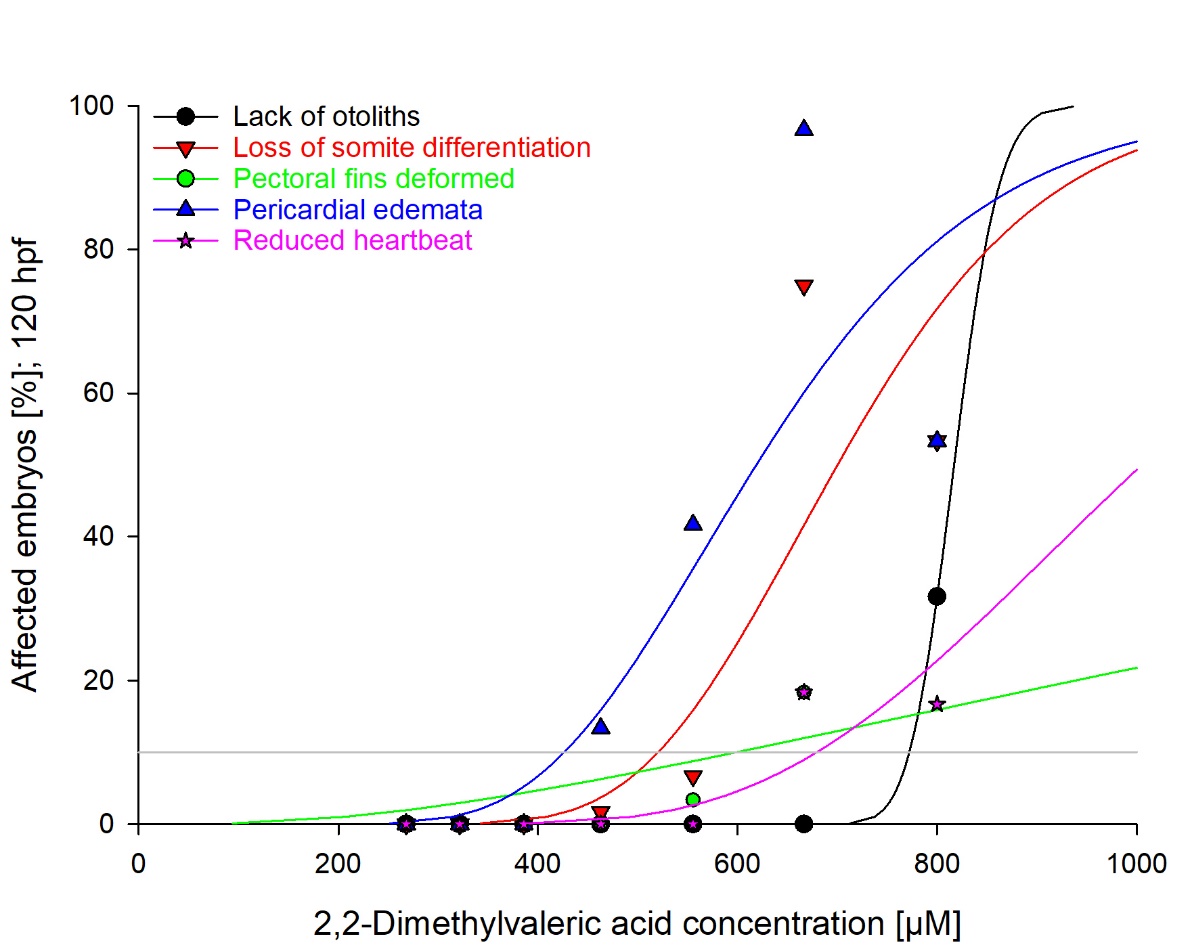


EC_10_ (µM)

772

520

599

426

679


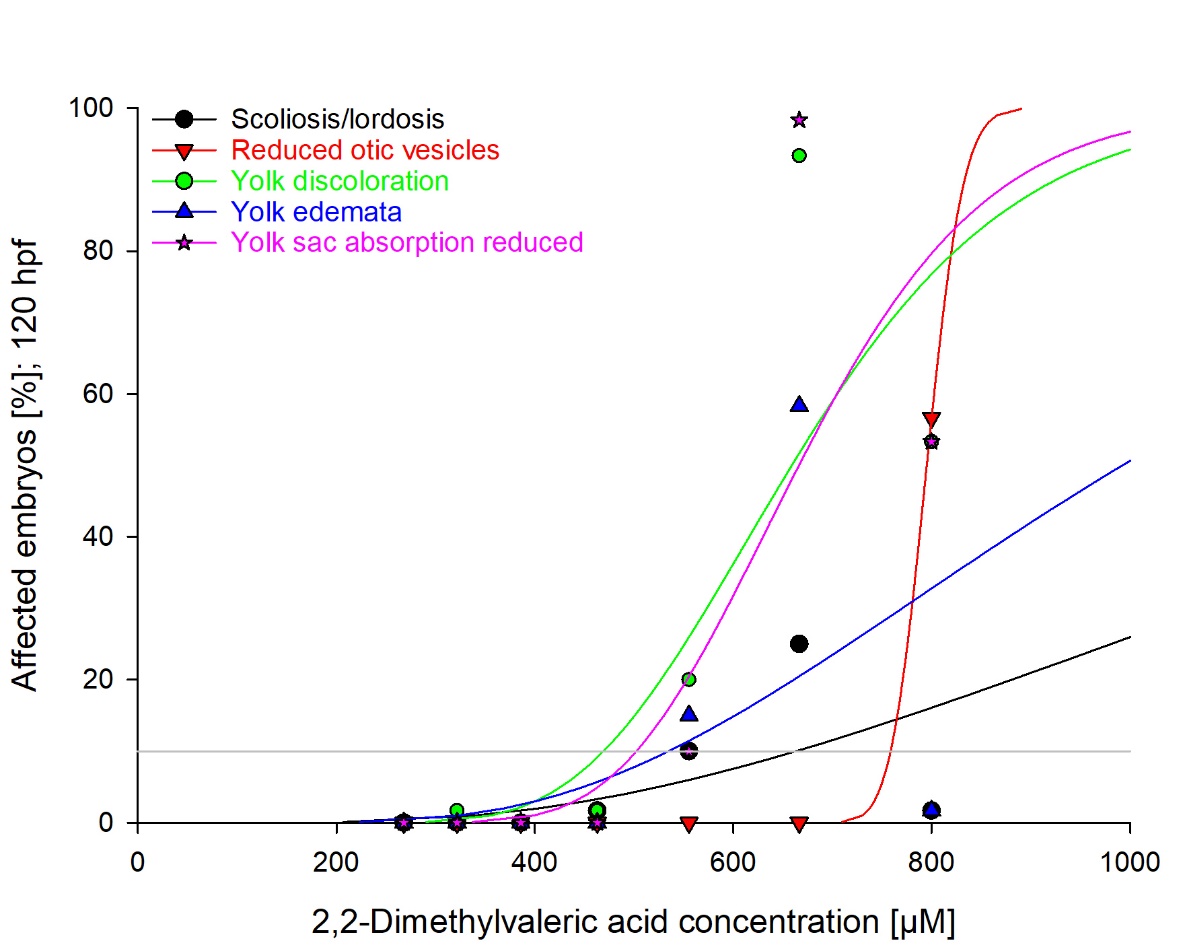


EC_10_ (µM)

663

759

469

535

502

Fig. S6 (continued): Dose-response curves for specific effects induced by exposure of zebrafish (*Danio rerio*) embryos to 2,2-dimethylvaleric acid for 120 h. Data are given as percentage of individuals affected (%) in n = 20 - 40 replicates.


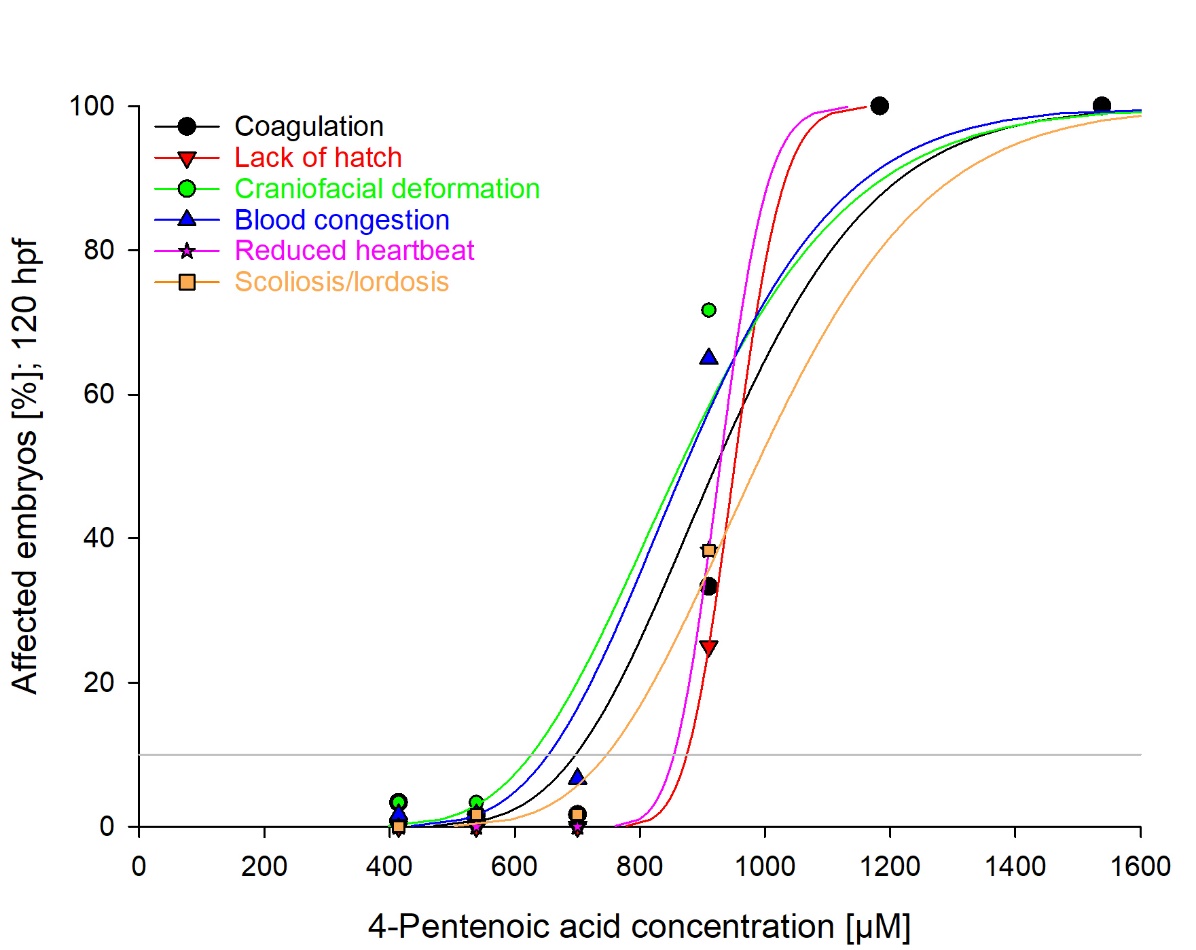


EC_10_ (µM)

697

875

626

654

854

747


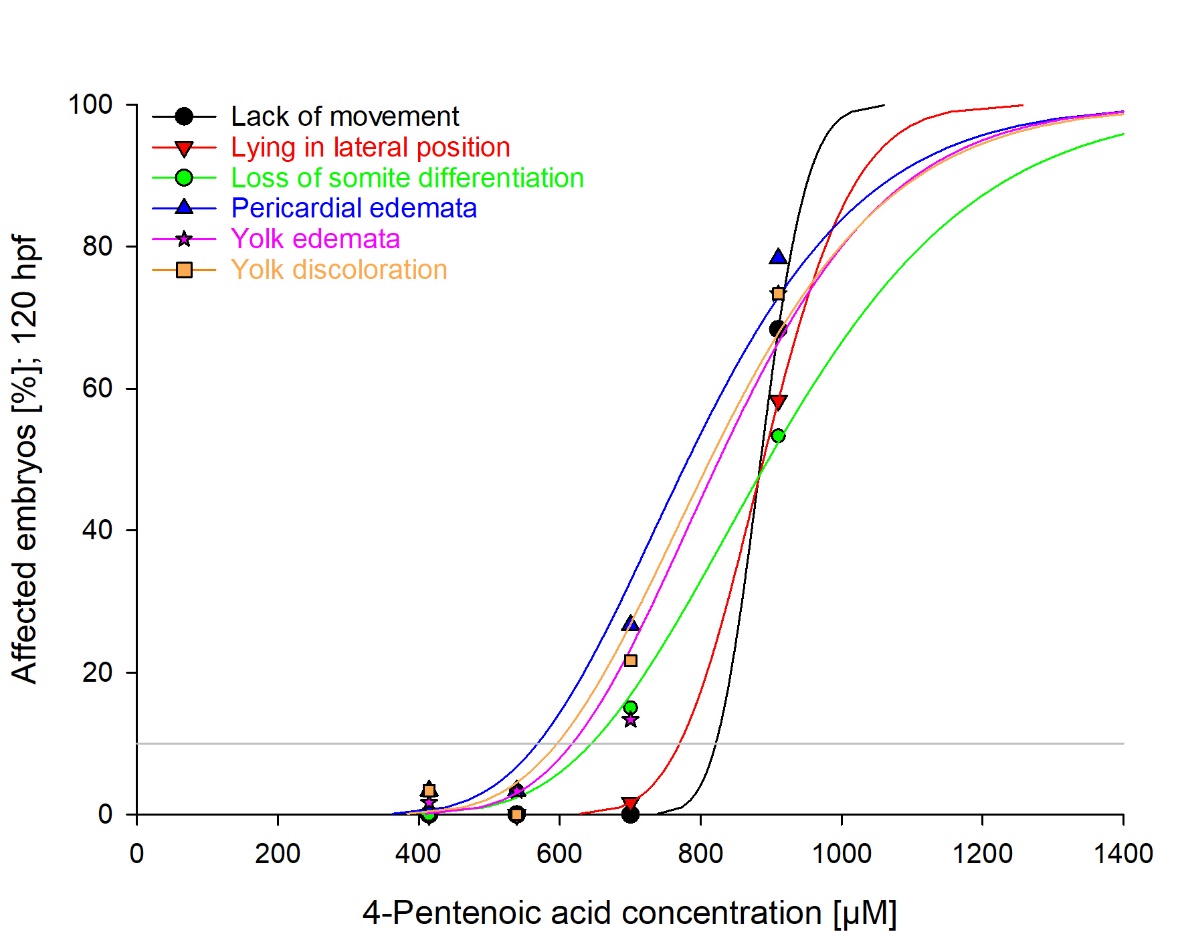


EC_10_ (µM)

822

770

645

568

618

596

Fig. S7: Dose-response curves for specific effects induced by exposure of zebrafish (*Danio rerio*) embryos to 4-pentenoic acid for 120 h. Data are given as percentage of individuals affected (%) in n = 20 - 40 replicates


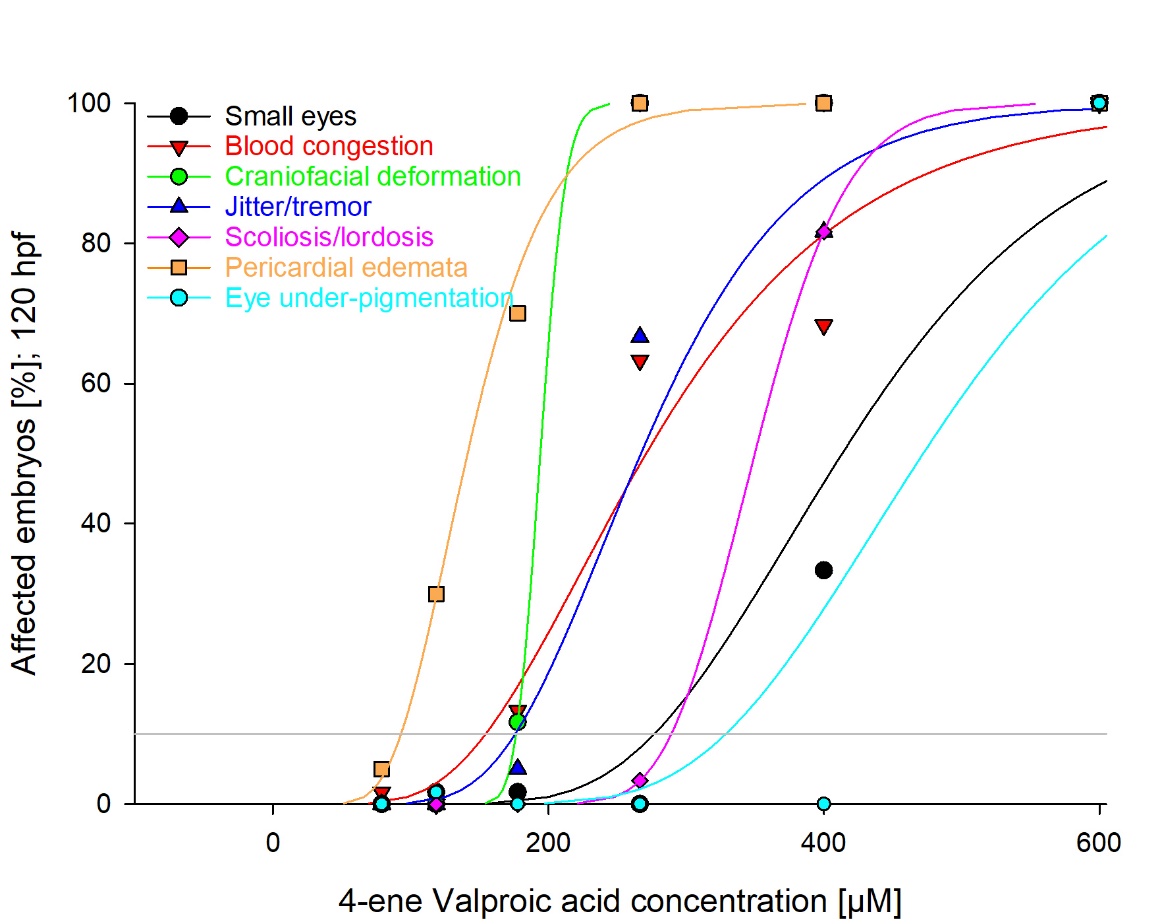


EC_10_ (µM)

277

154

177

176

289

93

329


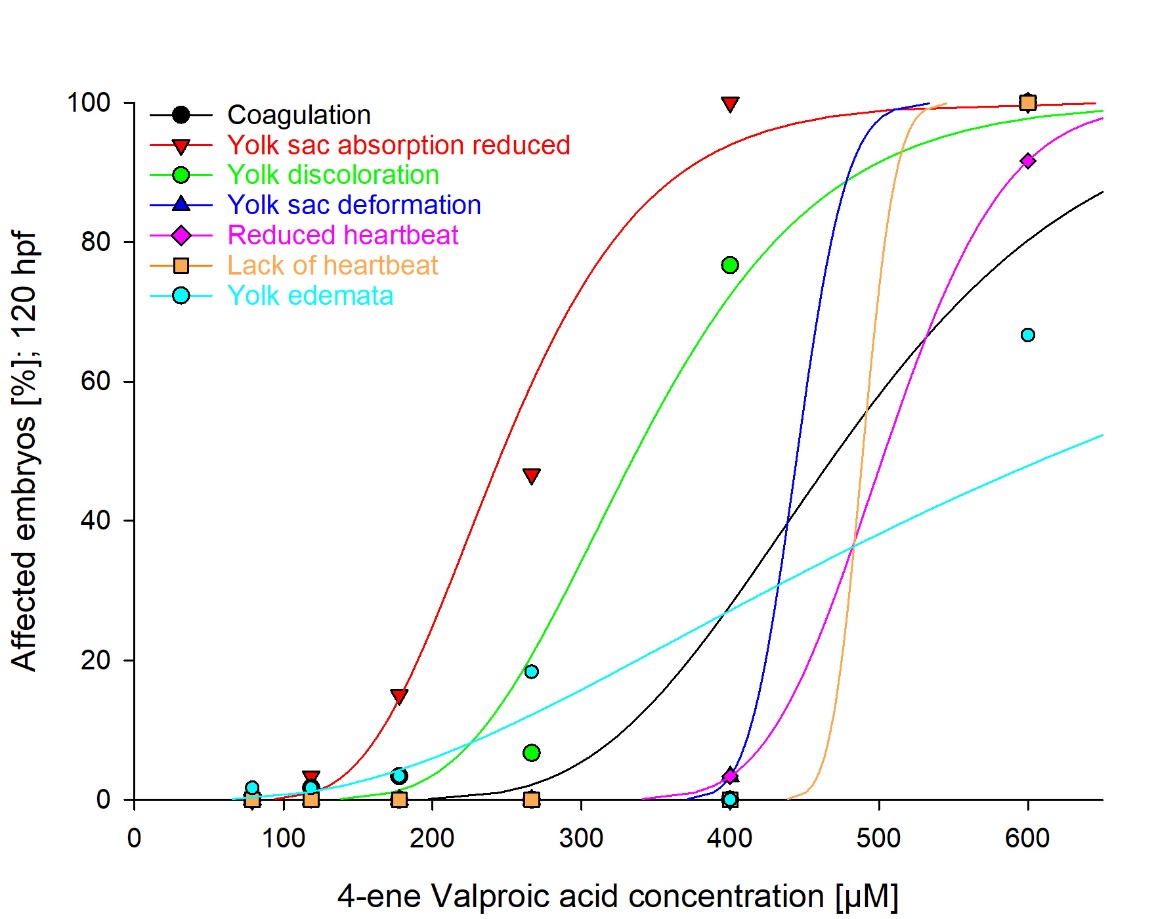


EC_10_ (µM)

328

166

233

413

429

468

245

Fig. S8: Dose-response curves for specific effects induced by exposure of zebrafish (*Danio rerio*) embryos to 4-*ene*-valproic acid for 120 h. Data are given as percentage of individuals affected (%) in n = 20 - 40 replicates.


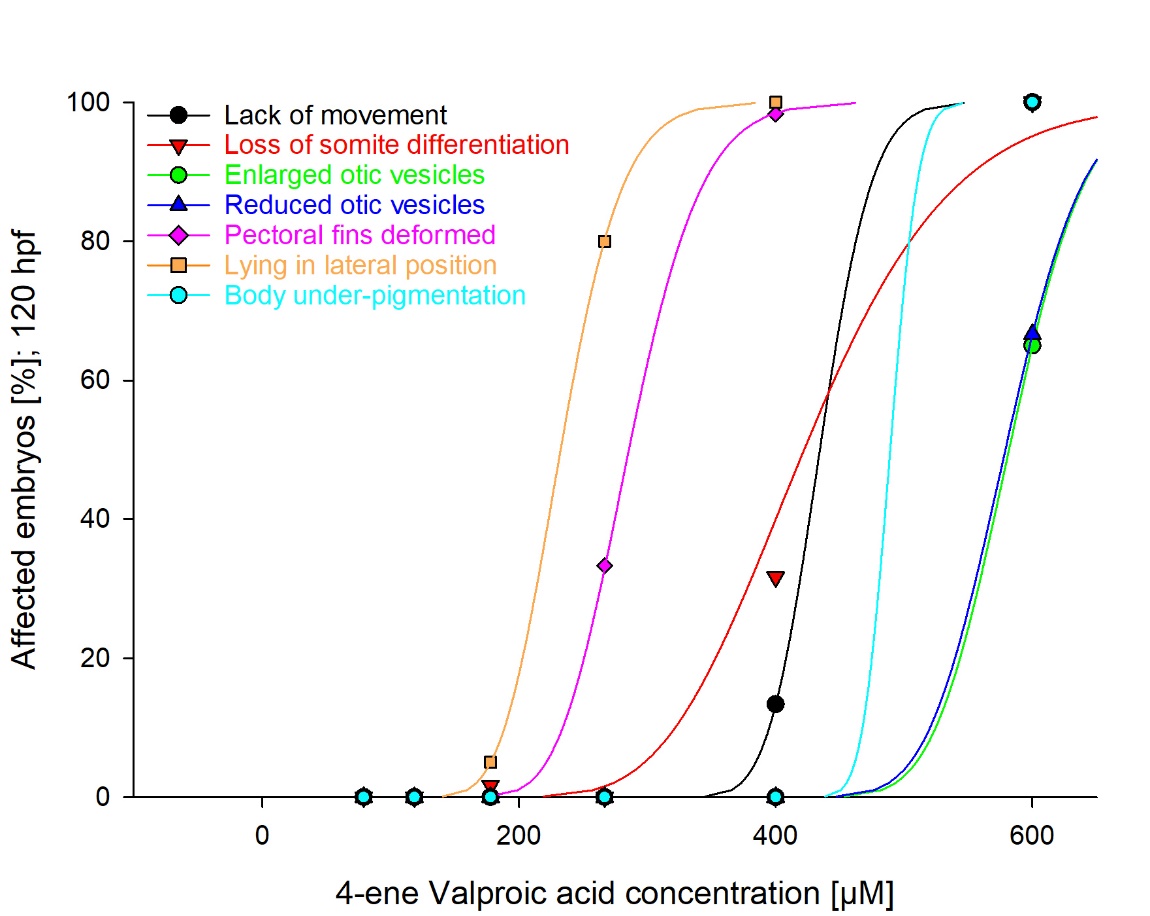


EC_10_ (µM)

395

322

525

520

234

189

468

Fig. S8 (continued): Dose-response curves for specific effects induced by exposure of zebrafish (*Danio rerio*) embryos to 4-*ene*-valproic acid for 120 h. Data are given as percentage of individuals affected (%) in n = 20 - 40 replicates.


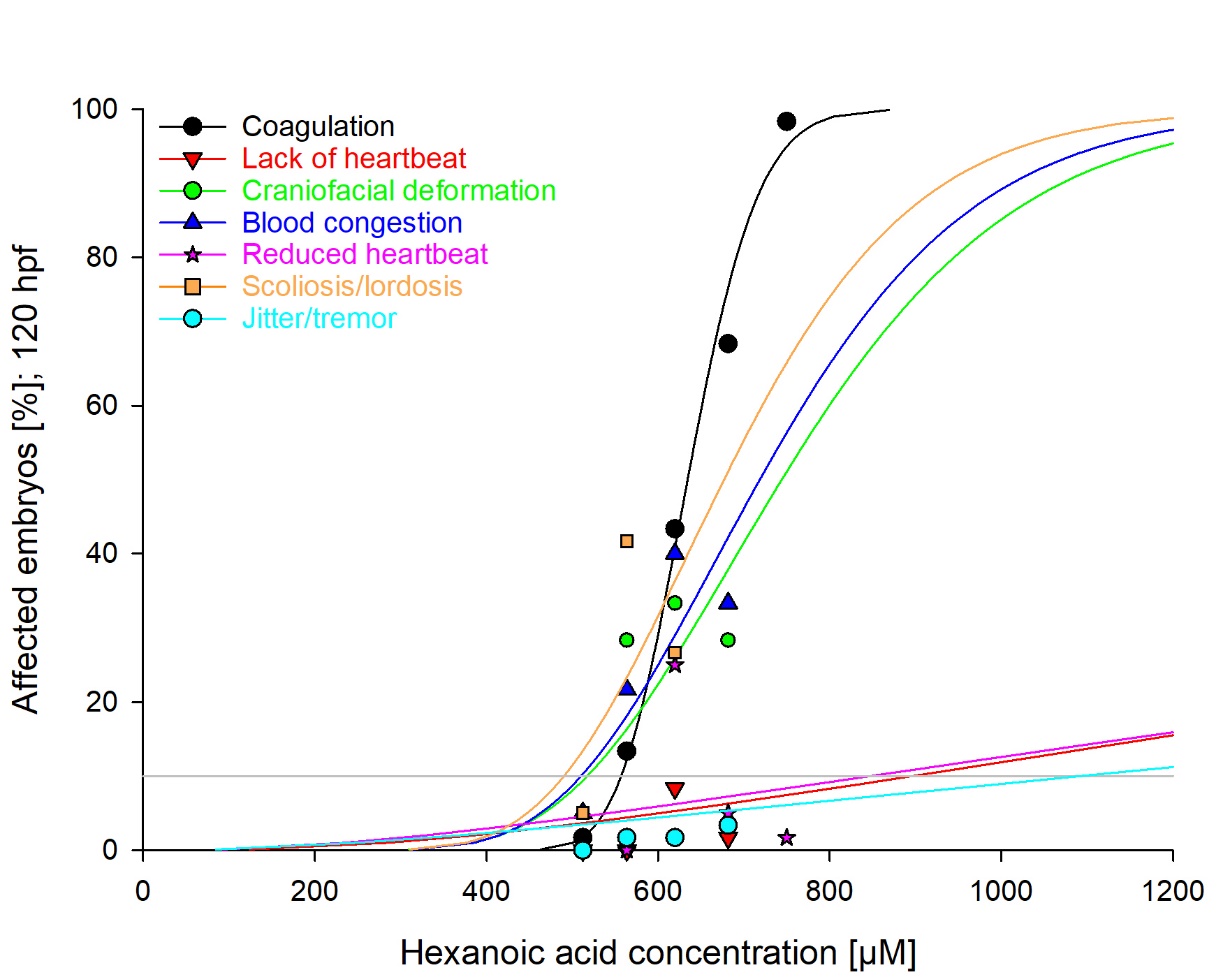


EC_10_ (µM)

557

897

517

510

848

490

1092


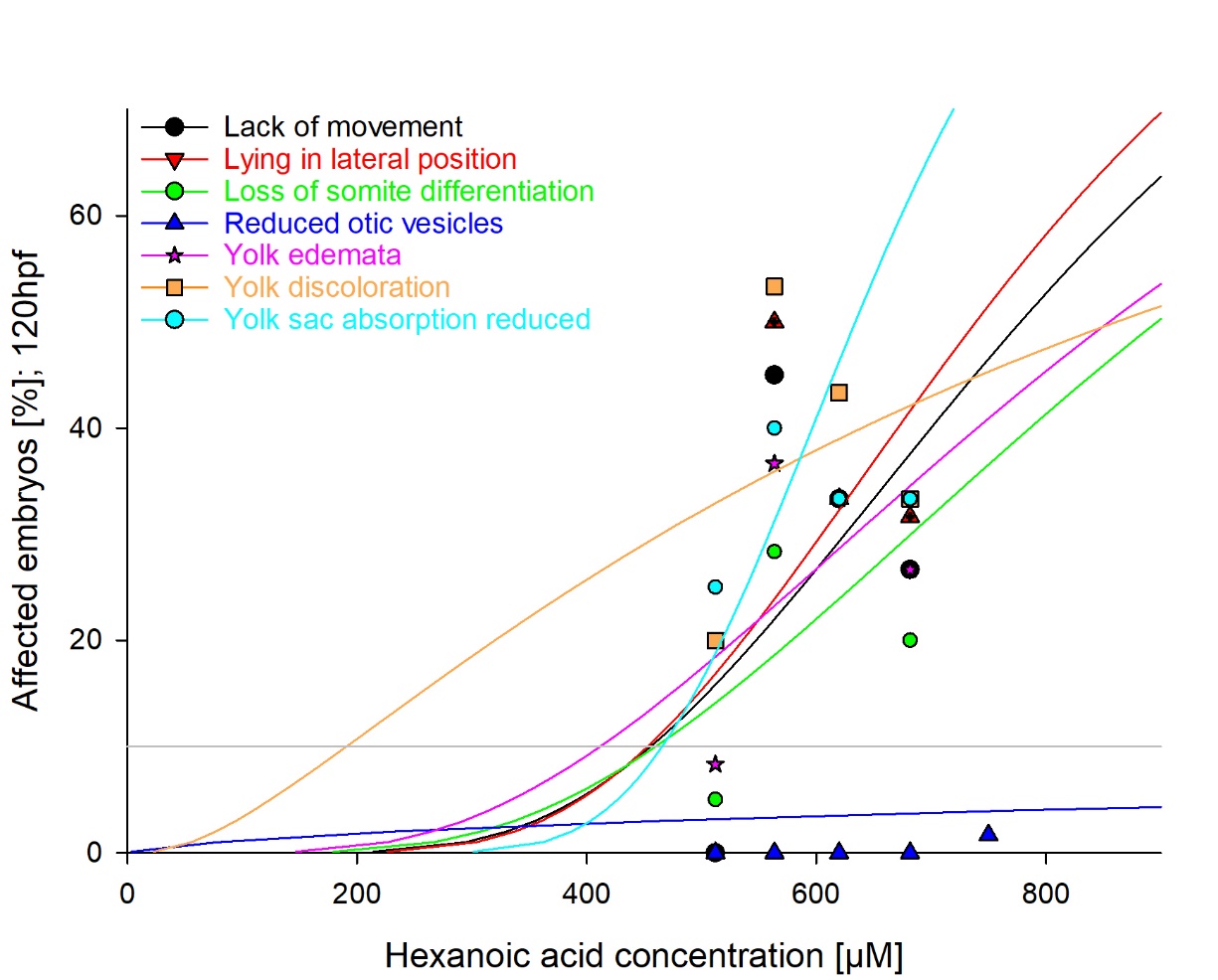


EC_10_ (µM)

456

453

460

n.d.

412

191

466

Fig. S9: Dose-response curves for specific effects induced by exposure of zebrafish (*Danio rerio*) embryos to hexanoic acid for 120 h for 120 h. Data are given as percentage of individuals affected (%) in n = 20 - 40 replicates.
